# Late Pleistocene pelagic gastropods of southern Taiwan: paleobiodiversity, first fossil records, and regional affinity

**DOI:** 10.7717/peerj.21046

**Published:** 2026-05-01

**Authors:** Diana Osipova, Chien-Hsiang Lin

**Affiliations:** 1 Biodiversity Research Center, Academia Sinica, Taipei, Taiwan; 2 Biodiversity Program, Taiwan International Graduate Program, Academia Sinica and National Taiwan Normal University, Taipei, Taiwan; 3 Department of Life Science, National Taiwan Normal University, Taipei, Taiwan

**Keywords:** Quaternary, Species dispersal, Biogeography, New records, Szekou Formation, Indo-Pacific

## Abstract

Holoplanktonic gastropods (pteropods and heteropods) are major components of modern Indo–West Pacific (IWP) plankton, yet their fossil record in this region remains sparse. Expanding the spatial and temporal coverage of fossil data is essential for reconstructing dispersal pathways of pelagic fauna within the IWP and understanding the origins of present-day diversity. Here, we describe a newly discovered Late Pleistocene assemblage of pelagic gastropods from southern Taiwan. The assemblage comprises 14 pteropod and eight heteropod taxa, most representing the first fossil records of holoplanktonic gastropods from Taiwan. We also evaluate variation in paleobiodiversity between depositional environments of the Szekou Formation. Species richness and density do not differ significantly between restricted and open lagoon settings, contrasting with patterns reported for benthic bivalves. To place these findings in a broader context, the newly reported assemblages were analyzed together with other Pleistocene assemblages across the IWP region. Only weak geographic and temporal separation was detected, suggesting a relatively cosmopolitan community composition in subtropical waters during the Pleistocene, likely reflecting low temperature variability despite glacial–interglacial cycles. Indicator species analysis further suggests a later arrival at higher latitudes for the pteropods *Telodiacria quadridentata* and *Heliconoides inflata*, which show associations with late Pleistocene sites, whereas *Styliola subula* displays a distribution resembling its modern range, being most closely associated with assemblages from Taiwan and southern Japan.

## Introduction

The distributional history of holoplanktonic shelled mollusks, represented by pteropods and heteropods, which independently adapted to a pelagic lifestyle, has been a subject of research for decades (Bernasconi & Robba, 1982; Shibata, 1986; Wall-Palmer et al., 2016; Janssen & Peijnenburg, 2017; Hart et al., 2020). Some of the main questions addressed in these studies concern the place of origin of both groups and their subsequent dispersal pathways. Pteropods are considered to have originated in the Atlantic during the middle to late Cretaceous (120–93 Myr and 93–66 Myr, respectively; Bernasconi & Robba, 1982; Janssen & Peijnenburg, 2017; Tajika, Nützel & Klug, 2018; Peijnenburg et al., 2020), whereas heteropods are thought to have appeared in the early Cretaceous, although their precise geographical origin remains uncertain (Van der Spoel, 1972; Wall-Palmer et al., 2016; Wall-Palmer et al., 2020).

Compared with the relatively well-documented fossil record of pelagic gastropods from the Atlantic (*e.g.*, reviewed in Wall-Palmer et al., 2016; Janssen & Peijnenburg, 2017; Peijnenburg et al., 2020), studies from the Indo–West Pacific (IWP) region remain patchy and uneven (discussed below). This stands in sharp contrast to the very high present-day biodiversity of these marine invertebrates in the IWP (*e.g.*, Janssen, Bush & Bednaršek, 2019), suggesting that large parts of the region are still understudied. Although the precise dispersal time and pathways of pelagic gastropods in the IWP are still debated, it is widely accepted that the global distribution of pteropods is strongly controlled by sea-surface temperature, salinity, and ocean circulation patterns (Hart et al., 2020). The distribution of extant taxa likely occurred relatively recently, perhaps during the Pliocene–Pleistocene (Bernasconi & Robba, 1982; Wall-Palmer et al., 2016; Janssen & Peijnenburg, 2017). The Pleistocene period (∼2.6 Ma), characterized by large amplitude glacial–interglacial variability, is believed to influence both the diversification and biogeographic distribution of pteropods (Janssen & Peijnenburg, 2017; Hart et al., 2020). Usually, the response to these changes is considered to be reflected in the morphological variation (Van der Spoel, 1962). At the same time, paleontological studies of heteropods have indicated that they were more tolerant of temperature fluctuations and were probably not significantly affected by changing climate conditions during the Quaternary (Van der Spoel, 1972; Wall-Palmer et al., 2014).

Since the early 20th century, most available fossil data from the IWP have come from Japan (summarized in Janssen, 2007), with more sporadic records from other localities, such as the Philippines (Janssen, 2007), Fiji Islands (Ladd, 1934; Janssen, 1999; Janssen & Grebneff, 2012), Indonesia (Janssen, 1999), and South Australia (Janssen, 1990). Fossil records of holoplanktonic gastropods from Taiwan are particularly scarce. Only two taxa of pteropods, *Clio mai* Chen & Huang, 1990 (Cavoliniidae) and *Limacina lini* Chen & Huang, 1990 (Limacinidae), have been reported from Oligocene deposits in northern Taiwan (Cheng, 1981; Chen & Huang, 1990). For a long time, these were the only available records. Recently, new occurrences have been reported from Late Pleistocene deposits in northern Taiwan (Osipova & Lin, 2025), highlighting the potential for a previously overlooked holoplanktonic gastropod fauna in Taiwan. In the present study, we (1) describe and analyze a recently discovered Late Pleistocene assemblage of pelagic gastropods from Taiwan and (2) assess how its paleobiodiversity compares with other reported Pleistocene assemblages from across the IWP region.

## Geological setting

**Figure 1:**
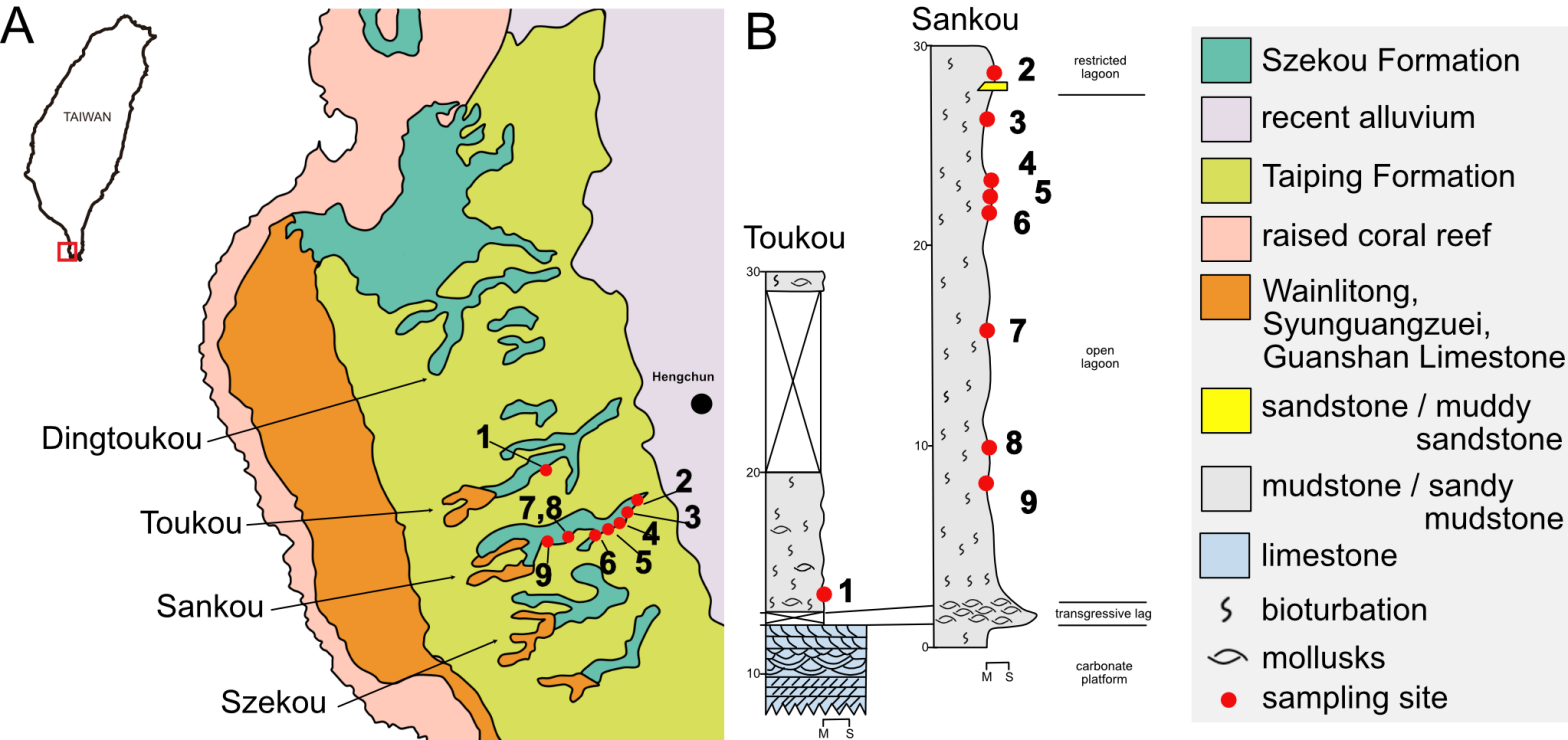
Geological and stratigraphic context of the Szekou Formation. (A) Geological map of the Szekou Formation with the distribution of sampling sites (adapted from Lin et al., 2022). (B) Stratigraphic columns of the Szekou Formation in Toukou and Sankou valleys (adapted from Chen et al., 1991).

The Szekou Formation is situated on the Hengchun West Platform, in the southwestern part of Taiwan (the Hengchun Peninsula). The formation occurs in small east–west–trending valleys, Dingtoukou, Toukou, Sankou, and Szekou (named from north to south; Fig. 1A), which dissect the platform (Cheng & Huang, 1975). The Szekou Formation overlies the Hengchun Limestone and is overlain by the Taiping Formation. It is primarily composed of silty to muddy, gray, firm mudstone, with the exposed thickness varying across localities, reaching up to 35 m (Chen et al., 1991). This formation is particularly noteworthy for its abundance and diversity of fossil mollusks (Wang, 1983; Chen et al., 1991; Hu, 1991–1995), while also containing other invertebrate fossils (Hu, 1981; Hu, 1984; Fong, 1989) and fish (Lin et al., 2021; Lin et al., 2022).

The formation has been subdivided into three units, with the lower unit further divided into two subunits (Chen et al., 1991; Fig. 1B). Sediments in the lower and middle units represent an open-lagoon environment, dominated by silty sandstone and muddy siltstone, which yield the highest abundance of mollusk fossils. In contrast, the upper unit records a restricted-lagoon setting, characterized by muddy siltstone with reduced mollusk diversity (Chen et al., 1991).

The age of the Szekou Formation remains a topic of debate, as different dating methods have yielded divergent results. For instance, radiocarbon dating of mollusk fossils indicates an age of 44–31 ka (Wang, Peng & Chen, 1991), whereas a study applying electron spin resonance analysis on the same fossil group points to an age of 140 ka (Shih et al., 2002). Similar estimates were obtained from palynological analysis, indicating an age of 140–100 ka (Cheng, Huang & Liew, 1986). Based on all lines of evidence, the Szekou Formation can be confidently regarded as Late Pleistocene in age.

## Materials & Methods

### Sediment sampling and specimen processing

A total of 69 bulk sediment samples, weighing over 345 kg in total, were collected during the dry seasons of 2022–2023 from nine sites within the Szekou Formation (Fig. 1A; Table S1). These included one outcrop at Toukou (site 1, *n* = 7) and eight outcrops at Sankou (sites 2–9, *n* = 62). Each sample was dried and weighed, after which 5 kg of sediment from each was set aside for subsequent diversity analyses. The sediment was disaggregated in water and wet-sieved through a 500 µm mesh. The residue was examined under a stereomicroscope, and shells of pelagic gastropods were carefully hand-picked. The total number of specimens recovered per site is summarized in Table 1, Table S2. The exact number of specimens examined for each taxon is listed in Table S3. Selected specimens were photographed using a digital camera adapted to a Nikon SMZ1270 stereomicroscope (Nikon, Tokyo, Japan), followed by creating stacked images using the Helicon Focus software (HeliconSoft, Kharkiv, Ukraine). To capture the finer structures and shapes of embryonic shells, we obtained scanning electron microscope (SEM) images of selected specimens.

**Table 1:** Number of specimens recovered from each sampling site.

**Taxon**	**Toukou open lagoon**	**Sankou restricted lagoon**	**Sankou open lagoon**	**Total**
	Site 1	Site 2	Sites 3–9	
*Atlanta helicinoidea*	0	0	1	1
*Atlanta rosea*	1	0	2	3
*Atlanta tokiokai*	1	0	2	3
*Atlanta vanderspoeli*	11	0	8	19
*Atlanta* cf. *peronii*	0	0	1	1
*Atlanta* sp.	2	0	4	6
*Oxygyrus inflatus*	1	0	3	4
*Protatlanta sculpta*	0	0	4	4
*Heliconoides inflatus*	10	0	27	37
*Creseis acicula*	13	0	9	22
*Styliola subula*	81	1	55	137
*Cuvierina* sp.	0	0	2	2
*Clio convexa*	1	0	1	2
*Clio pyramidata*	41	3	44	88
*Cavolinia globulosa*	1	0	0	1
*Cavolinia inflexa*	3	0	4	7
*Diacavolinia angulata*	9	1	39	49
*Diacavolinia bandaensis*	12	0	17	29
*Diacavolinia longirostris*	9	0	8	17
*Diacria erythra*	12	1	22	35
*Diacria trispinosa*	5	0	3	8
*Telodiacria quadridentata*	2	0	8	10
**Total**	215	6	264	**485**

Identifiable shells were classified to the lowest possible taxonomic level and included in quantitative analyses. Taxonomic identifications were based on relevant references. For each examined specimen, the following measurements were taken (Table S3): shell width (SW), shell height (SH), and aperture width (AW) and height (AH) for heteropods and pteropods with globose shells. Additional measurements included apical angle (AA) for *Atlanta vanderspoeli*, and rostrum length (RL) for pteropods possessing a rostrum.

All depicted specimens are deposited in the Biodiversity Research Museum, Academia Sinica, Taipei, Taiwan, under the prefix ASIZF, with each catalog number corresponding to a single specimen.

### Diversity measures

To examine potential differences in diversity and abundance between different depositional environments (open *vs.* restricted lagoons) of the Szekou Formation, retrieved specimens of pelagic gastropods were grouped into three assemblages: Toukou open-lagoon group (site 1), Sankou restricted-lagoon group (site 2), and Sankou open-lagoon group (sites 3–9).

Shell abundance (density) was calculated as the number of shells per kilogram of dry sediment for each sample. Taxonomic richness was expressed as the number of identified taxa per kilogram of dry sediment. Both abundance and richness data were visualized using box plots. Differences in specimen abundance and taxonomic richness among sites were tested using the Kruskal–Wallis test (Kruskal & Wallis, 1952), a non-parametric method suitable for comparing two or more groups. These analyses were performed in R version 4.4.2 (R Core Team, 2025) using the packages *rstatix* (Kassambara, 2023) and *ggpubr* (Kassambara, 2020) for statistical testing and visualization.

Diversity was quantified using Hill numbers (Hill, 1973) for three orders: ^0^*D* (species richness), ^1^*D* (Shannon diversity), and ^2^*D* (Simpson diversity). Because observed Hill numbers are sensitive to sampling completeness, we applied specimen-based rarefaction and extrapolation to standardize coverage and generate comparable diversity estimates (Chao & Jost, 2012). Rarefaction curves and 95% confidence intervals (based on 300 bootstrap replicates) were generated using the package *iNEXT* (Gotelli & Colwell, 2011; Chao et al., 2014; Hsieh, Ma & Chao, 2016). Data manipulation was carried out using packages *dplyr* (Wickham et al., 2025) and *readr* (Wickham, Hester & Bryan, 2024), and visualizations were produced with *ggplot2* (Wickham, 2016).

### Compositional analysis

To evaluate the relationship between the newly discovered Szekou assemblage and other known Pleistocene holoplanktonic gastropod assemblages from the IWP, we compiled a comprehensive dataset of previously reported occurrences (Table S4). The final dataset included 26 localities spanning Japan, Taiwan, and sediment core records from the Andaman Sea and Indian Ocean (Table S5). Previous studies have shown that the distribution of shallow-water marine mollusks reflects their thermal tolerances and can serve as an indicator of past climatic regimes (*e.g.*, Chinzei, 1986; Ogasawara, 1994). Quaternary glaciations strongly influenced the distribution of shallow-water genera, and during the Early and Middle Pleistocene a tropical climatic influence extended as far north as Shikoku and central Honshu, while regions farther north were characterized by temperate conditions (Ogasawara, 1994). Thus, localities from Japan were separated into two groups, southern and central Japan. Three time bins were defined: Early Pleistocene (Gelasian–Calabrian), Middle Pleistocene (Chibanian), and Late Pleistocene. Ages of localities were cross-checked and updated according to the most recent stratigraphic literature.

Taxonomic names were standardized using the WoRMS Taxon Match Tool (WoRMS Editorial Board, 2025). Where a taxon was reinterpreted in this work as representing a different taxon than that cited in the source, the reinterpreted taxon name was used. Additionally, taxa identified only at the genus level were treated as separate taxa from each locality. Occurrences assigned to ages outside the Pleistocene were excluded.

For comparisons among localities, occurrence data were converted into a presence–absence matrix. Pairwise similarities were calculated using the Jaccard index (Jaccard, 1912; Chao et al., 2005). Assemblages were then clustered using the unweighted pair-group method with arithmetic mean (UPGMA; Sokal & Michener, 1958) and, for comparison, complete linkage, both of which operate directly on Jaccard dissimilarities. Principal Coordinates Analysis (PCoA) was applied to the Jaccard matrix to visualize differences in assemblage composition. All analyses were conducted in R using the packages *vegan* (Oksanen et al., 2025) and *cluster* (Maechler et al., 2025).

Non-parametric one-way analysis of similarity (ANOSIM; Clarke, 1993) based on the Jaccard dissimilarity matrix was used to test for significant differences between predefined assemblage groups (age and locality). ANOSIM, which compares the mean of ranked dissimilarities within groups to those between groups, was performed in R using the *vegan* package. To identify taxa characteristic of particular assemblages, Indicator Species Analysis (ISA; Dufrêne & Legendre, 1997) was conducted using the package *indicspecies* (De Cáceres & Legendre, 2009).

## Results

### Systematic paleontology

Our synonymy list includes several original species descriptions that are not cited above: Benson (1835), Blainville (1821), Boas (1886), d’Orbigny (1935), Gray (1850), Issel (1911), Janssen (2003), Lesueur (1813), Linnaeus (1767), Quoy & Gaimard (1827), Rang (1828), Seapy, Lalli & Wells (2003), Souleyet (1852), Tokioka (1955), Van der Spoel (1970), Van der Spoel (1971), Van der Spoel & Troost (1972), Yamakawa & Ishikawa (1912), Zhang (1964).

**Table:** 

**Figure 2:**
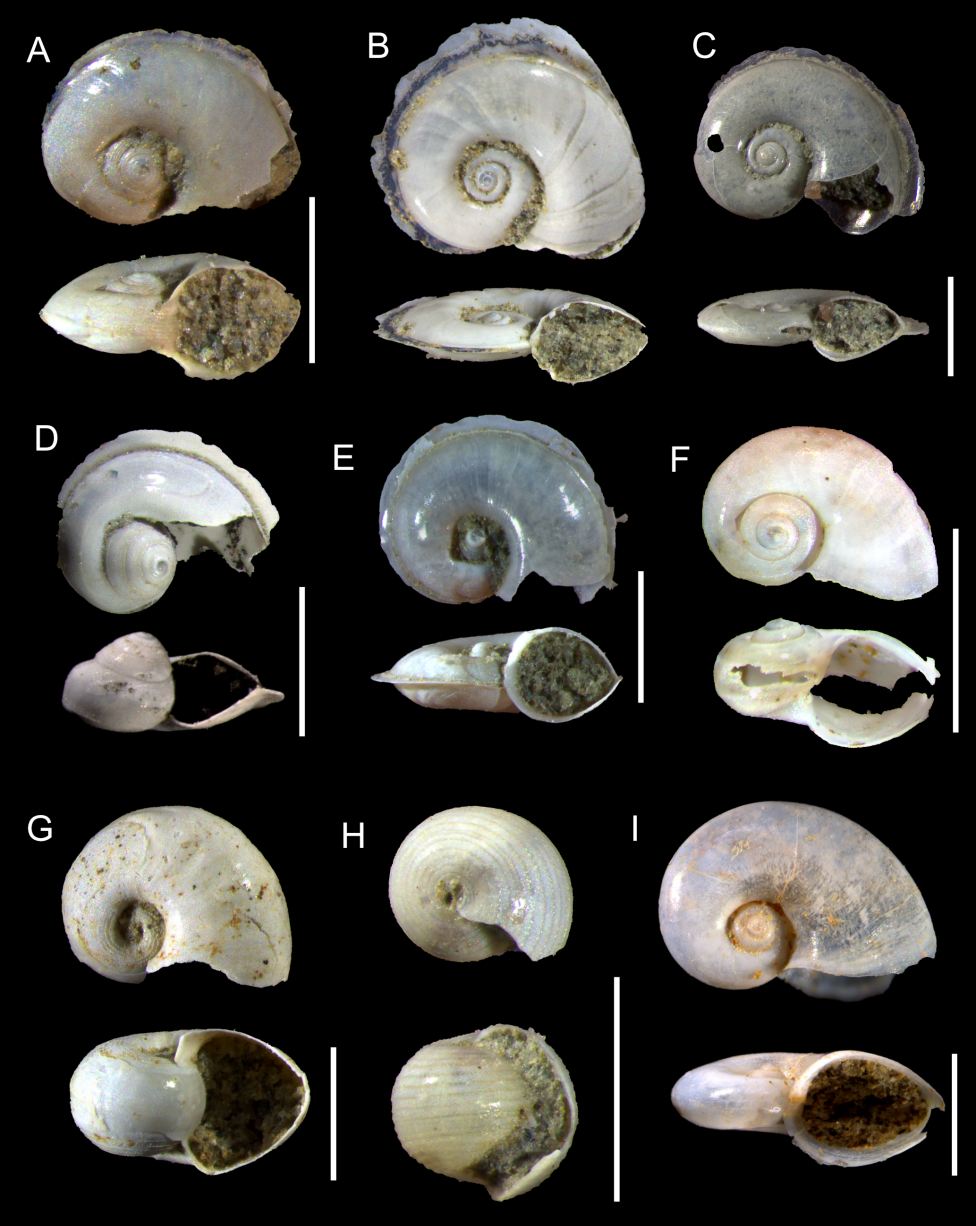
Species of the family Atlantidae from the Late Pleistocene Szekou Formation, Taiwan. (A) *Atlanta helicinoidea* Gray, 1850, ASIZF0101104. (B and C) *Atlanta rosea* Gray, 1850, ASIZF0101105–106. (D) *Atlanta tokiokai* Van der Spoel & Troost, 1972, ASIZF0101107. (E) *Atlanta vanderspoeli* Wall-Palmer, Hegmann & Peijnenburg, 2019, ASIZF0101108. (F) *Atlanta* cf. *peronii*, ASIZF0101109. (G and H) *Oxygyrus inflatus* (Benson, 1835), ASIZF0101110–111. (I) *Protatlanta sculpta* (Issel, 1911), ASIZF0101112. Scale bars for A, B–C, D, E, F, G, H, I equal 1 mm.

**Figure 3:**
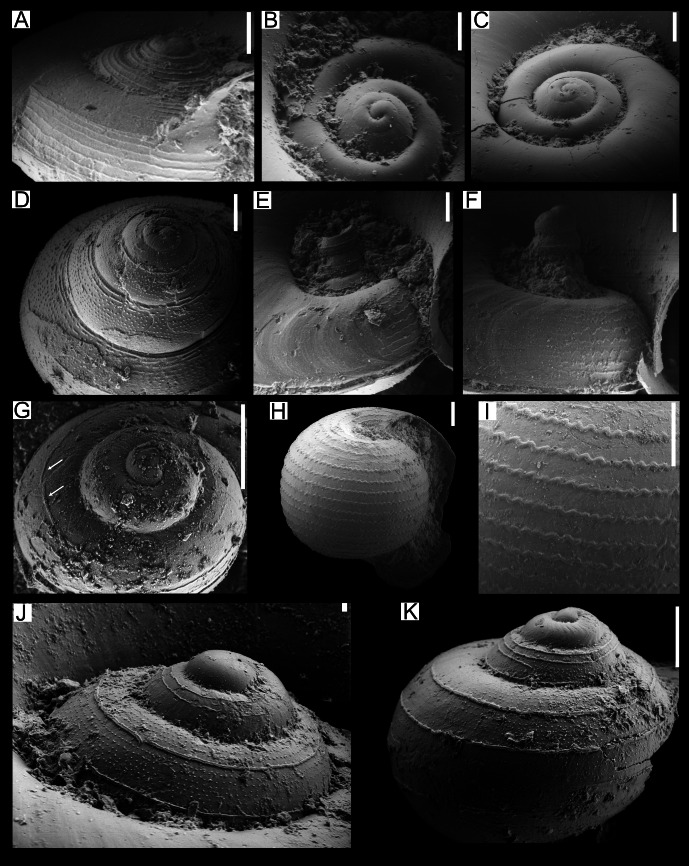
SEM images of species of the family Atlantidae from the Late Pleistocene Szekou Formation, Taiwan. (A) *Atlanta helicinoidea* Gray, 1850, ASIZF0101104. (B and C) *Atlanta rosea* (Gray, 1850), ASIZF0101105–106. (D) *Atlanta tokiokai* Van der Spoel & Troost, 1972, ASIZF0101107. (E and F) *Atlanta vanderspoeli* Wall-Palmer, Hegmann & Peijnenburg, 2019, (E) ASIZF0101108, (F) ASIZF0101113. (G)* Atlanta* cf. *peronii*, ASIZF0101109, arrows indicate spiral ringe. (H and I) *Oxygyrus inflatus* Benson, 1835, ASIZF0101111. (J and K) *Protalanta sculpta* Issel, 1911, (J) ASIZF0101112, (K) ASIZF0101114. Scale bars for A–I, K qual 100 µm, for J equal 10 µm.

Class Gastropoda Cuvier, 1795
Order Littorinimorpha Golikov & Starobogatov, 1975
Superfamily Pterotracheoidea Rafinesque, 1814
Family Atlantidae Rang, 1829
Genus *Atlanta* Lesueur, 1817
*Atlanta helicinoidea* (Gray, 1850)
(Figs. 2A, 3A)
1850 *Atlanta helicinoidea*; Gray: 101.
1983 *Atlanta heliconoides* Souleyet, 1852 non Gray, 1850; Shibata & Ujihara: 156, pl. 47, fig. 3.

**Description:** Shell laterally flattened, with five whorls regularly increasing in size. Shell surface differs in pattern with last whorl being smooth. Other whorls ornamented with spiral ridges. Spire wide, low, only slightly projecting over the shell plane. Sutures deep, but hard to determine due to ornamentation. Protoconch with one whorl, no ornamentation. Aperture subcircular. Keel poorly preserved.

**Remarks:** This species is represented by only one specimen from the studied locality. It can be easily distinguished from other atlantids from the Szekou Formation due to its ornamented shell with a broad and low spire. As a fossil from the IWP, this species was reported only from Late Pleistocene deposits of central Japan (Shibata & Ujihara, 1983).

**Table:** 

*Atlanta rosea* (Gray, 1850)
(Figs. 2B–2C, 3B–3C)
1850 *Atlanta rosea*; Gray: 101, pl. 241, fig. 2.

**Description:** Shell laterally flattened, medium (2.1 mm in SW, Table S3) with 4–5 whorls. Largest specimen with 5 whorls. Keel tall, appears on the last whorl of teleoconch. Shell surface smooth, shiny, with noticeable growth lines. Spire short, slightly projecting over last whorl in aperture view, slightly inclined. Sutures deep starting from second whorl of teleoconch. Protoconch small with three whorls, teleoconch whorl expands rapidly. Aperture oval narrowing toward outer margin.

**Remarks:** Shells belonging to the so-called *Atlanta peronii* species group (Richter & Seapy, 1999; Wall-Palmer et al., 2016) are relatively common in the sediments of the Szekou Formation. In some specimens, however, the initial whorls are damaged, which hinders precise taxonomic identification; such specimens are therefore classified as *Atlanta* sp. In recent adult specimens of *A. rosea*, the keel is inserted between the last and penultimate whorls, whereas this condition was not observed in the examined fossil material, suggesting that our specimens may represent immature individuals. Several closely related species inhabit modern waters of the IWP region. Among these, *A. rosea* can be distinguished by the shallow sutures of the first two whorls that smooth the protoconch into a nearly globular shape.

To date, this species has not been reported as a fossil from Asia. Janssen (2007) illustrated two specimens under the name *Atlanta oligogyra* Tesch, 1906 from the Pliocene deposits of Luzon, Philippines. However, one specimen (pl. 11, fig. 3) resembles the shape of the protoconch of those in *A. rosea*, and differs from another specimen (pl. 11, fig. 2). On the other hand, the last third whorl of two specimens increases rapidly in width, which is not common for *A. rosea*. Thus, this makes our record the first documented fossil occurrence of *A. rosea* from this region. Nonetheless, a re-examination of previously described material may reveal additional occurrences of this taxon.

**Table:** 

*Atlanta tokiokai* Van der Spoel & Troost, 1972
(Figs. 2D, 3D)
1972 *Atlanta tokiokai*; Van der Spoel & Troost: 2, fig. 1–3.
2007 *Atlanta tokiokai* Van der Spoel & Troost, 1972; Janssen: 50, pl. 15, fig. 4, fig. 5, pl. 16, fig. 1.

**Description:** Shell laterally flattened, medium in size (2.1 mm in SW, Table S3) with 5–5.5 whorls. Keel not tall, covers body whorl and placed between protoconch and body whorl. Suture shallow on protoconch, deep when the teleoconch starts. Shell surface smooth and glossy. Spiral lira slightly above the whorl periphery (Fig. 3D). Spire tall, cone shaped, inclined. Protoconch with 4 whorls, swollen.

**Remarks:** *Atlanta tokiokai* is known as a fossil only from Pliocene deposits in Luzon, Philippines (Janssen, 2007). This species has been reported to closely resemble *Atlanta inclinata* (Gray, 1850), but differs primarily in possessing a larger protoconch and a denser arrangement of tubercles on the spire surface (Fig. 3D). In the Luzon specimens, spiral lirae were described, with the second lira developing into the keel in adult individuals. This feature, however, was not observed in our material.

**Table:** 

*Atlanta vanderspoeli* Wall-Palmer, Hegmann & Peijnenburg, 2019
(Figs. 2E, 3E–3F)
2019 *Atlanta vanderspoeli*; Wall-Palmer, Hegmann & Peijnenburg: 73, figs. 7, 8.

**Description:** Shell small, around 1.6 mm in SW (Table S3). Spire slender and high with four whorls, protrudes over body whorls, apical angle of 40°. Sculpture of spiral ridges present up to fourth whorl. Keel well-developed on penultimate whorl. Edge of keel usually broken. Aperture sub-circular, narrowing toward keel.

**Remarks:** One of the most common species of heteropods in the Szekou Formation (Table 1). This species belongs to the *A. brunnea* species group (Wall-Palmer et al., 2019), which also includes *Atlanta brunnea* Gray, 1850 and *Atlanta turriculata* d’Orbigny, 1836. These three species are morphologically very similar, but can be reliably distinguished by the apical angle of the larval shell of 35–46° in *A. vanderspoeli,* which is wider than in *A. turriculata* but narrower than in *A. brunnea*. Our measurements (Table S3) yielded an average apical angle of 40°, supporting the identification of the Szekou specimens as *A. vanderspoeli*.

In modern oceans, this species is distributed across the Pacific, but no previous fossil records are known. Thus, the Szekou material represents the first fossil occurrence of *A. vanderspoeli*. However, distinguishing this species from its congeners can be challenging when preservation is poor, and it is possible that some fossil records historically attributed to *A. turriculata* may in fact represent *A. vanderspoeli*.

**Table:** 

*Atlanta* cf. *peronii*
(Figs. 2F, 3G)

**Description:** Shell laterally flattened, with 3.5 whorls. Shell surface smooth, shiny, with noticeable growth lines. Spire short, slightly projecting. Sutures deep. Protoconch small with three whorls, teleoconch whorl expands rapidly. Spiral lira on teleoconch whorls. Aperture oval narrowing toward outer margin.

**Remarks:** The only specimen showing features of *Atlanta peronii* Lesueur, 1817 was retrieved from the Szekou sediments. The specimen’s shell appears smooth without any noticeable pattern, with only a barely visible spiral rib being present on the spire (Fig. 3G, indicated by arrows). This feature was noted by Janssen (2007) and Frontier (1966). However, due to the fact that only one shell was identified and probably represents the juvenile specimen, it is hard to conclude the identification more precisely.

**Table:** 

*Atlanta* sp.

**Description:** Shell laterally flattened, medium in size (1.8 mm in SW, Table S3) with 3.5–4.5 whorls. Shell surface smooth, shiny, with noticeable growth lines. Spire with protoconch broken. Sutures deep. Aperture oval.

**Remarks:** Specimens are represented by *A. peronii*-like shells, which lack protoconch features. As long as one of the best distinguishing features is the depth of sutures on the protoconch, it is hard to estimate precisely whether these specimens belong to *Atlanta peronii* or *A. rosea*.

**Table:** 

Genus *Oxygyrus* Benson, 1835
*Oxygyrus inflatus* Benson, 1835
(Figs. 2G–3H, 3H, 3I)
1835 *Oxygyrus inflatus*; Benson: 175.
2003 *Oxygyrus keraudreni* (Lesueur, 1817); Seapy, Lalli & Wells: 537, fig. 18.
2007 *Oxygyrus keraudreni* (Lesueur, 1817); Janssen: 51, pl. 1, figs. 2, 3; pl. 16, figs. 2–4.

**Description:** Shell involute, not flattened, with convex, rounded whorls. Teleoconch of one body whorl in the biggest specimen, increase in size rapidly. Body whorl smooth, with visible growth lines. Protoconch with distinct ∼20 zig-zag, almost evenly spaced lirae. Aperture subcircular in bigger specimens, dorso-ventrally compressed closer to outer margin. Aperture of juveniles moon-shaped (arched), deeply sinuated.

**Remarks:** Specimens of *O. inflatus* can be readily recognized by the distinctive ornamentation of the protoconch. Within the Szekou assemblage, *O. inflatus* is rare; however, we recovered specimens that also preserve the teleoconch (Fig. 2G). Because the teleoconch is composed of conchiolin, its preservation in the fossil record is exceptional and therefore rarely observed. The transition from the ornamented protoconch to the smooth teleoconch has been described and illustrated by Thiriot-Quiévreux (1973) and Batten & Dumont (1976).

The only previously known fossil occurrence of this species in the IWP is from Pliocene deposits in Luzon, Philippines (Janssen, 2007), where it was reported under the name *Oxygyrus keraudreni* (Lesueur, 1817). Janssen (2007) also documented juveniles bearing an ornament-free “belt” near the ventral shell periphery. This feature was not observed in our material, possibly because the studied specimens represent earlier ontogenetic stages. Together with the Philippine record, our findings constitute the second fossil occurrence of *O. inflatus* in the IWP region.

**Table:** 

Genus *Protatlanta* Tesch, 1908
*Protatlanta sculpta* Issel, 1911
(Figs. 2I, 3J–3K)
1911 *Protatlanta sculpta*; Issel: 3, figs. 1–5.
1983 *Protatlanta souleyeti* (Smith, 1888); Shibata & Ujihara: 154, pl. 46, fig. 1.
2007 *Protatlanta souleyeti* (Smith, 1888); Janssen: 54, pl. 17, fig. 4, pl. 18, figs. 1 2.

**Description:** Shell laterally flattened, medium in size (1.8 mm in SW, Table S3), with 4–4.5 whorls, without keel. Surface of teleoconch smooth, shiny, with noticeable growth lines. Spire moderately high, not projecting over last whorl in aperture view. Sutures deep. Protoconch of three whorls. Surface of protoconch with two thin spiral ribs above periphery. In between these ribs, seven to 10 rows of finer dotted lines. Separation of protoconch and teleoconch distinct as ornamentation disappears rapidly after third whorl. Fourth body whorl increase rapidly in width.

**Remarks:** This species was only recently confirmed as valid (Wall-Palmer et al., 2016), making it the second extant representative of the genus *Protatlanta*. However, because the taxonomic status of *P. sculpta* has been debated for several decades, some authors have identified it as *Protatlanta souleyeti*, particularly when dealing with fossil material. In modern oceans, *P. sculpta* is considered to occur exclusively in the Atlantic (Wall-Palmer et al., 2016; Wall-Palmer et al., 2018). By contrast, fossil records indicate its presence in the IWP, although these occurrences have generally been attributed to *P. souleyeti*. Notable examples include two occurrences from central and southern Japan, dated to the Middle and Late Pleistocene, respectively (Shibata & Ujihara, 1983), as well as several Pliocene occurrences from Luzon in the Philippines (Janssen, 2007). Moreover, in his abundant material from the Philippines, Janssen (2007) reported and suggested that two distinct shell morphotypes were present, likely representing both *P. souleyeti* and *P. sculpta*.

**Table:** 

**Figure 4:**
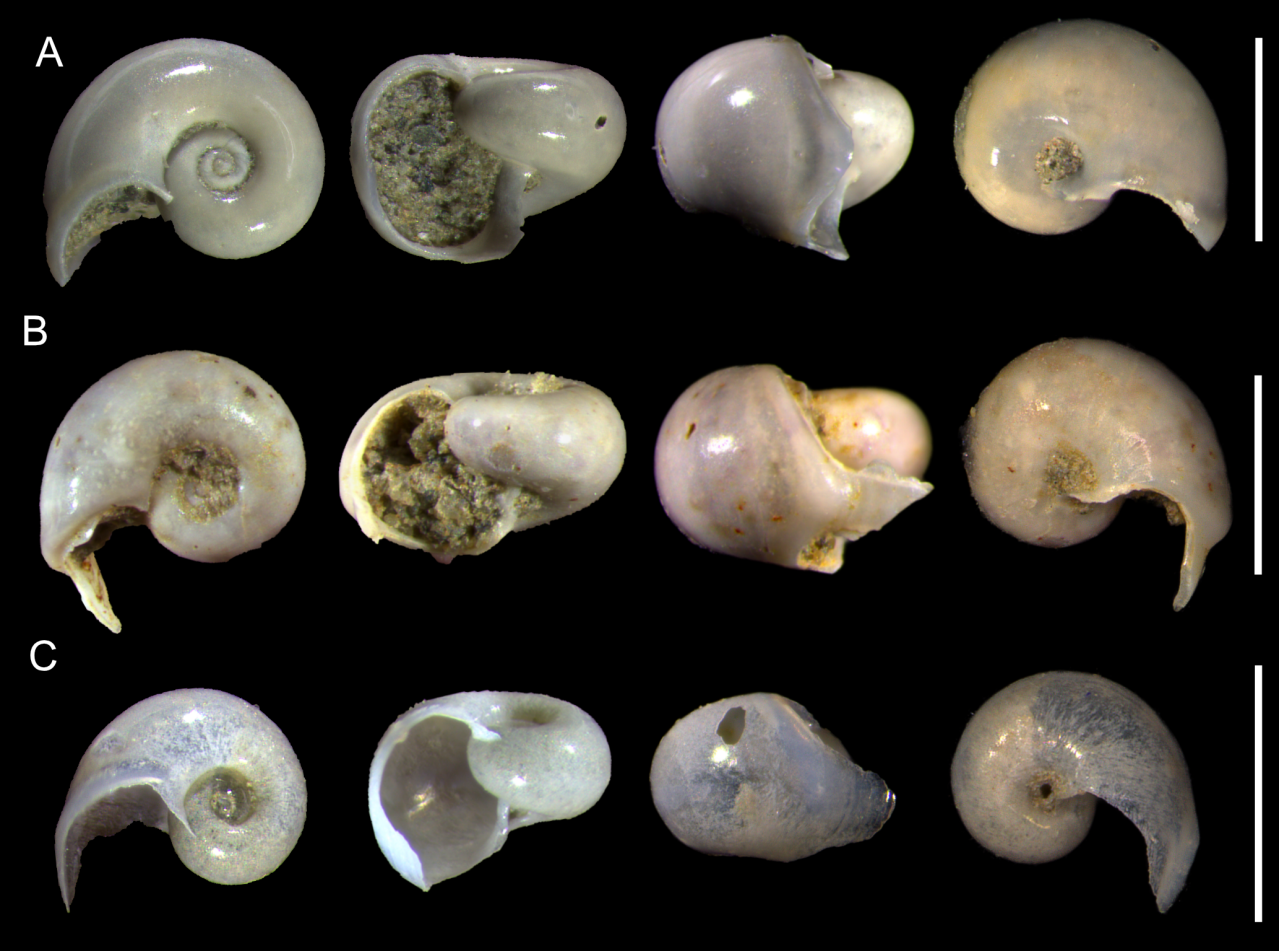
Species of the family Heliconoididae from the Late Pleistocene Szekou Formation, Taiwan. (A–C) *Heliconoides inflatus* (d’Orbigny, 1835), ASIZF0101115–117. Scale bars equal 1 mm.

Order Pteropoda Cuvier, 1804
Suborder Euthecosomata Meisenheimer, 1905
Superfamily Limacinoidea Gray, 1840
Family Heliconoididae Rampal, 2019
Genus *Heliconoides* d’Orbigny, 1835
*Heliconoides inflata* (d’Orbigny, 1835)
(Figs. 4A–4C)
1835 *Atlanta* (*Heliconoides*) *inflata*; d’Orbigny: 174, pl. 12, figs. 16–19.
1983 *Limacina inflata* (d’Orbigny, 1835); Shibata & Ujihara: 158, pl. 43, fig. 1.
1990 *Limacina inflata* (d’Orbigny, 1835); Janssen: 14, pl. 2, figs. 5–7, pl. 3, fig. 11, pl. 10, fig. 2.
1984 *?Limacina inflata* (d’Orbigny, 1835); Shibata: 78.
2003 *Heliconoides inflata* (d’Orbigny, 1835); Janssen: 168.
2007 *Heliconoides inflata* (d’Orbigny, 1835); Janssen: 60, pl. 2, figs. 1–3; pl. 21, figs. 1–3.

**Description:** Shell coiled, planorboid, sinistral, wider than high (0.7 mm in SH, 1.1 mm in SW, Table S3). Shell surface smooth and glossy. Adult specimens (Fig. 4A) with 4.5 whorls. Spire not wide and sunken. Whorls convex, last body whorl increases considerably in size and slightly flattened laterally closer to aperture. Evident circular thickening observed closer the aperture. Umbilicus deep, but not wide. Aperture wide and subcircular, height of aperture of the maximum shell height. Abapical part of aperture slightly bent outwards.

**Remarks:** This species is comparatively abundant in the Szekou Formation (Table 1), where it is represented by individuals at different developmental stages. In the western Pacific deposits, it is predominantly known from the Pliocene–Pleistocene. The oldest records from this region are Miocene specimens from Australia (Muddy Creek, Hamilton, and Altona Bay), described by Janssen (1990). In central Southeast Asia, *H. inflata* has been reported from Pliocene deposits in Luzon, Philippines (Janssen, 2007). Additional occurrences are documented from Pliocene–Pleistocene deposits in Japan, ranging from the Okinawa Islands (Shibata & Ujihara, 1983) to central Honshu (Shibata & Ujihara, 1983; Shibata, 1986).

**Table:** 

**Figure 5:**
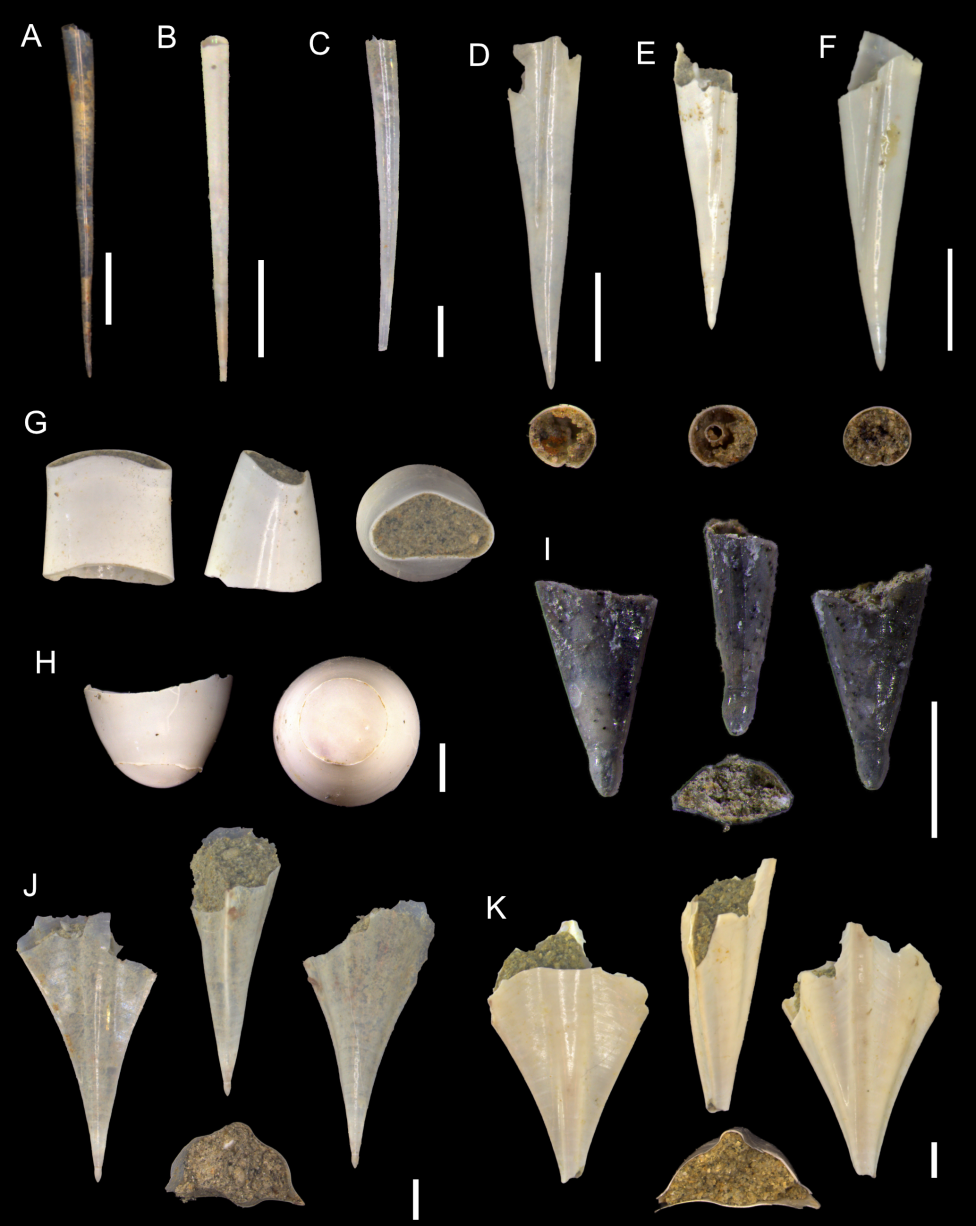
Species of families Creseidae, Cuvierinidae, and Cliidae from the Late Pleistocene Szekou Formation, Taiwan. (A–C) *Creseis acicula* (Rang, 1828), ASIZF0101118–120. (D–F) *Styliola subula* (Quoy & Gaimard, 1827), ASIZF0101121–123. (G and H) *Cuvierina* sp., ASIZF0101124–125. (I) *Clio convexa* (Boas, 1886), ASIZF0101126. (J and K) *Clio pyramidata* (Linnaeus, 1767), ASIZF0101127–128. Scale bars for A, B, C, D, E–F, G–H, I, J, K equal 1 mm.

**Figure 6:**
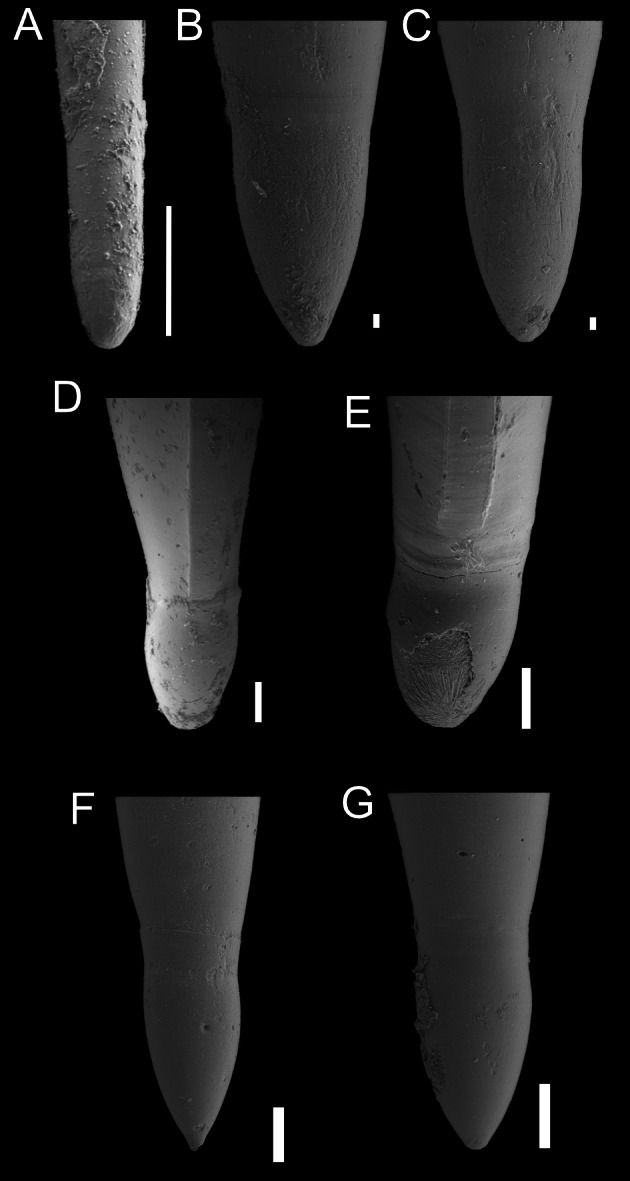
SEM images of species of the families Creseidae and Cliidae from the Late Pleistocene Szekou Formation, Taiwan. (A) *Creseis acicula* (Rang, 1828), ASIZF0101118. (B and C) *Styliola subula*, (B) ASIZF0101121, (C) ASIZF0101123. (D and E) *Clio convexa* (Boas, 1886), (D) ASIZF0101126, (E) ASIZF0101129. (F and G) *Clio pyramidata* Linnaeus, 1767, ASIZF0101130–131. Scale bars for A–B, E–H equal to 100 µm, for C–D equal to 10 µm.

Superfamily Cavolinioidea Gray, 1850 (1815)
Family Cavoliniidae Gray, 1850 (1815)
Family Creseidae Rampal, 1973
Genus *Creseis* Rang, 1828
*Creseis acicula* (Rang, 1828)
(Figs. 5A–5C, 6A)
1828 *Cleodora* (*Creseis*) *acicula*; Rang: 318, pl. 17, fig. 6.

**Description:** Shell elongated, needle-like with light uneven curvature along length. Width increases towards aperture. Aperture circular. Protoconch small with rounded tip.

**Remarks:** This species is prone to misidentification due to the strong similarity of its shell to those of several scaphopod groups. *Creseis acicula* can be distinguished from scaphopods, particularly representatives of the families Calliodentaliidae Chistikov, 1975, Rhabdidae Chistikov, 1975, and Gadilinidae Chistikov, 1975, by its considerably thinner shell. In addition, scaphopod shells typically exhibit distinct circular growth lines, which are absent in *C. acicula*. Specimens that preserve their protoconch can also be readily distinguished from scaphopods. The geological record of this species and related synonymy in the western Pacific region is discussed in Osipova & Lin (2025).

**Table:** 

Genus *Styliola* Gray, 1847
*Styliola subula* (Quoy & Gaimard, 1827)
(Figs. 5D–5F, 6B–6C)
1827 *Cleodora subula*; Quoy & Gaimard: 233–234, pl. 8D, figs. 1–3.

**Description:** Shell straight and conical. Longitudinal groove runs across shell in spiral way. Aperture suboval with compression created by longitudinal groove. Protoconch elliptical with pointed tip, visually divided from teleoconch shell by circular depression.

**Remarks:** This species is comparatively abundant within the Szekou holoplanktonic fauna (Table 1). Specimens show good preservation of almost the entire shell, especially the protoconch. The related synonymy and geological occurrences of this species in the West Pacific area are discussed in Osipova & Lin (2025).

**Table:** 

Family Cuvierinidae Van der Spoel, 1967
Genus *Cuvierina* Boas, 1886
*Cuvierina* sp.
(Figs. 5G–5H)

**Description:** Only aperture and caudal parts preserved. Shell closer to aperture compressed dorso-ventrally, depression deeper on ventral side. Aperture triangular in shape. In lateral view dorsal apertural lip higher. Caudal septum present, convex.

**Remarks:** The presence of only two fragmentary specimens of *Cuvierina* from the Szekou Formation prevents a more precise species-level identification. In fossil records of the Pacific region, the genus is best represented by *Cuvierina columnella* (Rang, 1827), which is the most abundant and widely distributed species. This taxon has been reported from multiple Pliocene–Pleistocene formations in Japan, ranging from the Okinawa Islands to Honshu (Shibata & Ishigaki, 1981; Shibata & Ujihara, 1983; Shibata, 1984). Additional species described from Pliocene deposits include *Cuvierina intermedia* (Bellardi, 1873) from Japan (Ujihara, 1996), as well as *Cuvierina tubulata* Collins, 1934 and *Cuvierina urceolaris* (Mörch, 1850) from the Philippines (Janssen, 2007) and Japan (Shibata, 1986; Ujihara, Shibata & Saito, 1990). Among these, *C. tubulata*, currently regarded as *Cuvierina astesana* (Janssen, 2006), and *C. intermedia* are considered extinct, as they are absent from younger Pleistocene deposits. Morphological features further support this distinction: *C. astesana* and *C. urceolaris* possess more reniform apertures, while *C. intermedia* is characterized by a concave upper portion of the shell’s lateral outlines. These traits are inconsistent with the Szekou material, suggesting that the specimens may instead be attributable to *C. columnella*.

**Table:** 

Family Cliidae Jeffreys, 1869
Genus *Clio* Linnaeus, 1767
*Clio convexa* (Boas, 1886)
(Figs. 5I, 6D–6E)
1886 *Cleodora pyramidata* var. *convexa;* Boas: 73, 203, pl. 6, figs. 97a–d.
2007 *Clio convexa convexa* (Boas, 1886); Janssen: 79–81, pl. 3, fig. 13; pl. 4, figs. 1, 3; pl. 24, figs. 6, 7.

**Description:** Shell slightly convex, almost straight, pyramidal, triangular in cross-section. Greatest width at aperture. Surface glossy. Teleoconch slightly flattened antero-posteriorly. Doubled carinae start right after constriction separating protoconch and teleoconch. Protoconch separated by distinct constriction, bulged, tip rounded.

**Remarks:** Only two specimens were recovered from the Szekou Formation. Both lack the upper part of the teleoconch, preventing the description of additional diagnostic features. Nevertheless, the most distinctive characteristic of this species, which allows its differentiation from other *Clio* species in the Szekou Formation, is the presence of carinae on the lateral sides of the shell, beginning immediately after the separation from the protoconch (Bé & Gilmer, 1977; Janssen, 2007). These carinae are paired and exhibit a square shape in transverse section (Janssen, 2007). In recent Pacific Ocean waters, *Clio polita* Pelseneer, 1888 has been recorded (*e.g.*, Janssen, Bush & Bednaršek, 2019), and it also possesses paired carinae. Young or fragmented specimens of *C. convexa* in our material can be distinguished from *C. polita* by the shape of the protoconch, which is globular in *C. polita*.

*Clio convexa* has previously been reported from a sediment core from the Andaman Sea, dated to the Late Pleistocene (Sijinkumar, Nath & Guptha, 2010). The only other published occurrence of this species is from the Pliocene deposits of Pangasinan, Luzon (Janssen, 2007). Janssen (2007) noted frequency differences between *C. convexa* and *Clio pyramidata* (Linnaeus, 1767) in the Pangasinan material. Although the two species co-occurred in most samples, *C. pyramidata* was more abundant in some samples, whereas *C. convexa* dominated in others. In the studied Szekou samples, *C. pyramidata* is more frequent than *C. convexa*, following the same trend observed in the Pangasinan assemblage.

**Table:** 

*Clio pyramidata* Linnaeus, 1767
(Figs. 5j–5K, 6F–6G)
1767 *Clio pyramidata*; Linnaeus: 1094.
1912 *Clio pyramidata* Linnaeus, 1767; Yamakawa & Ishikawa: 4, pl. 1, fig. 5.
1955 *Euclio pyramidata lanceolata* (Lesueur, 1813); Tokioka: 62, pl. 8, figs. 11–13.
1964 *Euclio pyramidata lanceolata* (Lesueur, 1813); Zhang: 141, fig. 16.
1979 *Euclio pyramidata lanceolata* Lesueur, 1813; Shibata: pl. 20, figs. 9–18.
1983 *Clio pyramidata lanceolata* (Lesueur, 1813); Shibata & Ujihara: 162, pl. 44, figs. 5, 8. 1984 *Clio pyramidata* (Linnaeus, 1767); Shibata: 81, pl. 24, figs. 1–3.
1984 *Clio* (*Clio*) *pyramidata* forma *lanceolata* (Lesueur, 1813); Shibata: 81, pl. 24, figs. 1–3.
1986 *Clio pyramidata* (Linnaeus, 1767); Shibata, Ishigaki & Ujihara: 47, pl. 7, figs. 5, 6.
1986 *Clio* (*Clio*) *pyramidata* forma *lanceolata* (Lesueur, 1813); Shibata, Ishigaki & Ujihara: 47, pl. 7, figs. 5, 6.
1990 *Clio pyramidata* forma *lanceolata* (Lesueur, 1813); Ujihara, Shibata & Saito: 315, pl. 1, figs. 10, 11.
2007 *Clio* (*Clio*) *pyramidata* forma *lanceolata* (Lesueur, 1813); Janssen: 81–82, pl. 4, fig. 4; pl. 24, figs. 9, 10.

**Description:** Shell straight, pyramidal, triangular in cross section. Greatest width at aperture. Surface glossy. Teleoconch tube-shaped first 1.5 mm, then widens rapidly. Dorsal side convex, with three radial ribs. Central rib evident when shell increases in width, in cross section protrudes slightly above aperture. Lateral ribs wider. Ventral side concave with one central ridge. Growth lines clearly defined. Protoconch separated by distinct constriction, bulged, tip pointed.

**Remarks:** This species is comparatively common within the Szekou assemblage (Table 1). Most specimens consist of early teleoconchs with well-preserved protoconchs. *Clio pyramidata* exhibits a wide range of morphological variation, and its different forms have been treated as distinct species, subspecies, or morphotypes by various authors (reviewed in Janssen et al., 2018).

Two forms commonly identified in Neogene–Quaternary deposits of the Pacific region are *Clio pyramidata* forma *pyramidata* Linnaeus, 1767, and *Clio pyramidata* forma *lanceolata* (Lesueur, 1813). Of the two, *C. pyramidata* forma *lanceolata* is thought to have had a slightly wider geographical distribution (Tesch, 1913; Rampal, 2002; Janssen, 2007) and to differ morphologically by having more concave lateral margins than *C. pyramidata* forma *pyramidata* (Janssen, 2007). These diagnostic features are observable only in larger specimens (Shibata, 1984). Both forms are reported from Japanese deposits ranging from the Miocene to the Pleistocene (Shibata & Ishigaki, 1981; Shibata & Ujihara, 1983; Shibata, 1984; Shibata, 1986; Ujihara, Shibata & Saito, 1990; Ujihara, 1996). In addition, *C. pyramidata* forma *lanceolata* has also been documented from Pliocene deposits in Luzon. The examined material from Szekou largely consists of small or fragmentary shells, which makes it difficult to assign them to a particular form with confidence. Differences from other *Clio* species within the Szekou assemblage are discussed above.

**Table:** 

**Figure 7:**
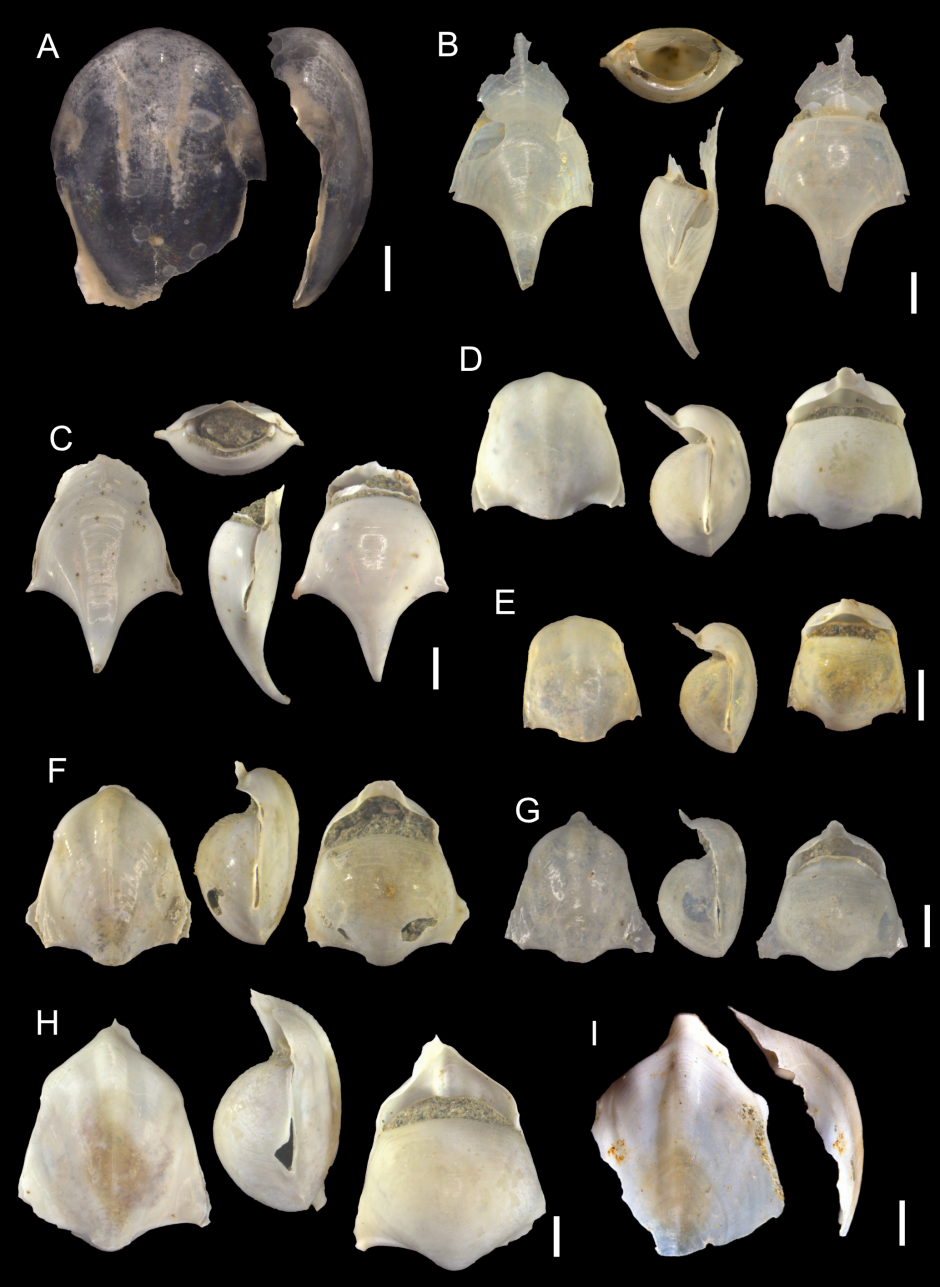
Species of the subfamily Cavoliniinae from the Late Pleistocene Szekou Formation, Taiwan. (A) *Cavolinia globulosa* Gray, 1850, ASIZF0101132. (B and C) *Cavolinia inflexa*, ASIZF0101133–134. (D and E) *Diacavolinia angulata* (Souleyet, 1852), ASIZF0101135–136. (F and G) *Diacavolinia bandaensis* Van der Spoel, Bleeker & Kobayasi, 1993, ASIZF0101137–138. (H and I) *Diacavolinia longirostris* (Blainville, 1821), ASIZF0101139–140. Scale bars for A, B, C, D–E, F–G, H, I equal 1 mm.

Family Cavoliniidae Gray, 1850 (1815)
Subfamily Cavoliniinae Gray, 1850 (1815)
Genus *Cavolinia* Abildgaard, 1791
*Cavolinia globulosa* Gray, 1850
(Fig. 7A)
1850 *Cavolinia globulosa*; Gray: 8.
2025 *Cavolinia globulosa* Gray, 1850; Osipova & Lin: 6, figs. 3A–B.

**Remarks:** Description of Pleistocene *C. globulosa* from Taiwan and related synonymy in Osipova & Lin (2025). From the Szekou assemblage, only a single specimen of *C. globulosa* was retrieved, representing a dorsal part of the shell. Previously, this species was reported from northern Taiwan Late Pleistocene deposits (Osipova & Lin, 2025).

**Table:** 

*Cavolinia inflexa* (Lesueur, 1813)
(Figs. 7B–7C)
1813 *Hyalaea inflexa*; Lesueur: 285, pl. 5, fig. 4.
1983 *Cavolinia inflexa labiata* (d’Orbigny, 1835); Shibata & Ujihara: 165, pl. 44, fig. 4, pl. 45, fig. 4.

**Description:** Shell elongated, slightly concave. Greatest width between two lateral spines (3.0 mm in SW, Table S3). Ventral side more inflated (swollen) than the dorsal, comprises almost all shell height in later view. Ventral margin of aperture lip bends upward with deep groove underneath it. Dorsal side flattened, slightly curved outwards. No radial ribs present. In some specimens, distinct arch-like growth lines on both sides closer to aperture. Dorsal aperture lip well developed, projecting beyond ventral lip. Lateral sides of dorsal lip slightly bent. Lateral spines wide and short. Apical part slightly depressed dorso-ventrally, curved in dorsal direction. Protoconch absent.

**Remarks:** Within the Szekou material, this species is rare, being represented by only a few well-preserved shells. It has been reported from several Pliocene–Pleistocene localities in Japan; however, in most sources, its occurrence is merely noted without accompanying description or illustration (*e.g.*, Shibata & Ishigaki, 1981; Shibata, 1986). The nominal species *Cavolinia inflexa* (*sensu stricto*) is known from only a limited number of localities. Specimens previously identified as *Cavolinia inflexa labiata* (d’Orbigny, 1835), now recognized as a distinct species *Cavolinia labiata*, have been recovered from Pleistocene deposits in the Okinawa Islands and Honshu (Shibata & Ujihara, 1983). *C. labiata* differs from *C. inflexa* by having a broader shell. Another morphotype, *Cavolinia inflexa* forma *kakegawaensis* (Shibata, 1984), was reported from Pleistocene sediments in central Honshu (Shibata, 1984; Ujihara, 1996). Representatives of this form are characterized by the presence of three distinct dorsal ribs. Based on these differences, we can conclude that our Szekou specimens are representatives of *C. inflexa*.

**Table:** 

Genus *Diacavolinia* Van der Spoel, 1987
*Diacavolinia angulata* (Souleyet, 1852)
(Figs. 7D–7E)
1852 *Hyalaea angulata*; Souleyet: 152, pl. 5 figs. 1–6.
1984 *Cavolinia longirostris* forma *angulosa* (Gray, 1850); Shibata: 87, pl. 25, fig. 7.
1983 *Cavolinia longirostris angulosa* (Gray, 1850); Shibata & Ujihara: 165, pl. 45, fig. 6.

**Description:** Shell slightly inflated, triangular. Greatest width within lateral spines (2.7 mm in SW, Table S3). Ventral side gradually well-inflated, comprises two-thirds of shell height in lateral view. Transverse riblets slightly visible closer to aperture. Ventral apertural margin gradually curved, slightly bent outward. Aperture narrow, moon-shaped, almost covered by dorsal aperture lip. Dorsal side slightly convex. Five radial ribs divided by narrow interspace. Rostrum with no constriction, separated from dorsal side by deep transverse groove, creating hump. Notch present on one specimen. Central rib protrudes slightly over hump. Transverse ornament closer to outer hump. Outer hump well developed, fully visible from apertural view, from dorsal view almost straight. Lip bellies apparent, lip shoulders well developed. Lateral spines formed by extensions of ventral shell part, dorsally shorter, slightly bent outwards. Protoconch shaded, opening closed without septum, but narrow slit might be visible.

**Remarks:** Specimens of *D. angulata* are one of the most abundant among the Szekou assemblage (Table 1). Distinguishing characters compared to closely related species are discussed below. The only reported occurrences of this species from the Pacific area are from Japanese Pliocene–Pleistocene deposits (Shibata & Ujihara, 1983; Shibata, 1984; Shibata, 1986), usually as subspecies or forma of *Diacavolinia longirostris* (Blainville, 1821).

**Table:** 

*Diacavolinia bandaensis* Van der Spoel, Bleeker & Kobayasi, 1993
(Figs. 7F–7G)
1993 *Diacavolinia bandaensis*; Van der Spoel, Bleeker & Kobayasi: 144, figs. 19A–B, pl. 2, fig. 24.
2025 *Diacavolinia* cf. *bandaensis*; Osipova & Lin: 10, fig. 5A.

**Description:** Shell slightly inflated, triangular. Greatest width within lateral spines (3.6 mm in SW, Table S3). Ventral side moderately convex, comprises two-thirds of shell height in lateral view. Transverse riblets slightly visible closer to aperture. Ventral apertural margin gradually curved, slightly bent outward. Dorsal side smooth and glossy, flattened. Growth lines on the dorsal side. Five longitudinal ribs, central three the most evident, lateral ribs wide, separated from lateral spines by recession. Central rib longer than others. Rostrum separated from dorsal side by deep transverse groove, moderate in width, with no notch. Outer hump considerably arched from dorsal view. Lip gutter evident. Lip bellies slightly bent outwards, lip shoulder moderately developed. Aperture triangular, moon-shaped. Lateral spines formed by extensions of ventral shell part, dorsally shorter, slightly bent outwards. Protoconch shaded, opening closed without septum, but narrow slit might be visible.

**Remarks:** This species is less abundant compared to a closely related *D. angulata* (Table 1). The discussion on the main difference between *D. angulata* and *D. bandaensis* can be found in Van der Spoel, Bleeker & Kobayasi (1993). However, *D. bandaensis* can be readily distinguished from *D. angulata* by the noticeably arched hump from the dorsal view and slightly bigger shells (Table S3). Additionally, aperture height is bigger, and lip bellies are less bent outwards than in *D. angulata*.

Previously, this species was not known from the fossil record. One imperfect specimen was recovered from the Toukoshan Formation, northern Taiwan (Osipova & Lin, 2025), which is supposed to be the first fossil record. Our findings from the Szekou assemblage provide additional evidence of the existence of *D. bandaensis* at least from the Pleistocene in Asia.

**Table:** 

*Diacavolinia longirostris* (Blainville, 1821)
(Figs. 7H–7I)
1821 *Hyalaea longirostris*; Blainville: 81.
1912 *Cavolinia longirostris* (Blainville, 1821); Yamakawa & Ishikawa: 19, pl. 6, fig. 11;
1955 *Cavolinia longirostris longirostris* (Blainville, 1821); Tokioka: 67, pl. 9, figs. 22–22;
1964 *Cavolinia longirostris* (Blainville, 1821); Zhang: 150, fig. 23.
1970 *Cavolinia longirostris longirostris* (Blainville, 1821); Van der Spoel: 124, fig. 24;
1971 *Cavolinia longirostris longirostris* (Blainville, 1821); Van der Spoel: 15, fig. 20;
1983 *Cavolinia longirostris longirostris* (Blainville, 1821); Shibata & Ujihara: 164, pl. 45, fig. 5.
1984 *Cavolinia longirostris* forma *longirostris* (Blainville, 1821); Shibata: 86, pl. 25, figs. 5, 6.

**Remarks:** See description for the Late Pleistocene *D. longirostris* from Taiwan and related synonymy in Osipova & Lin (2025). Within the Szekou Formation, this species is rare, mostly being represented by fragmentary dorsal parts of the shell.

**Table:** 

**Figure 8:**
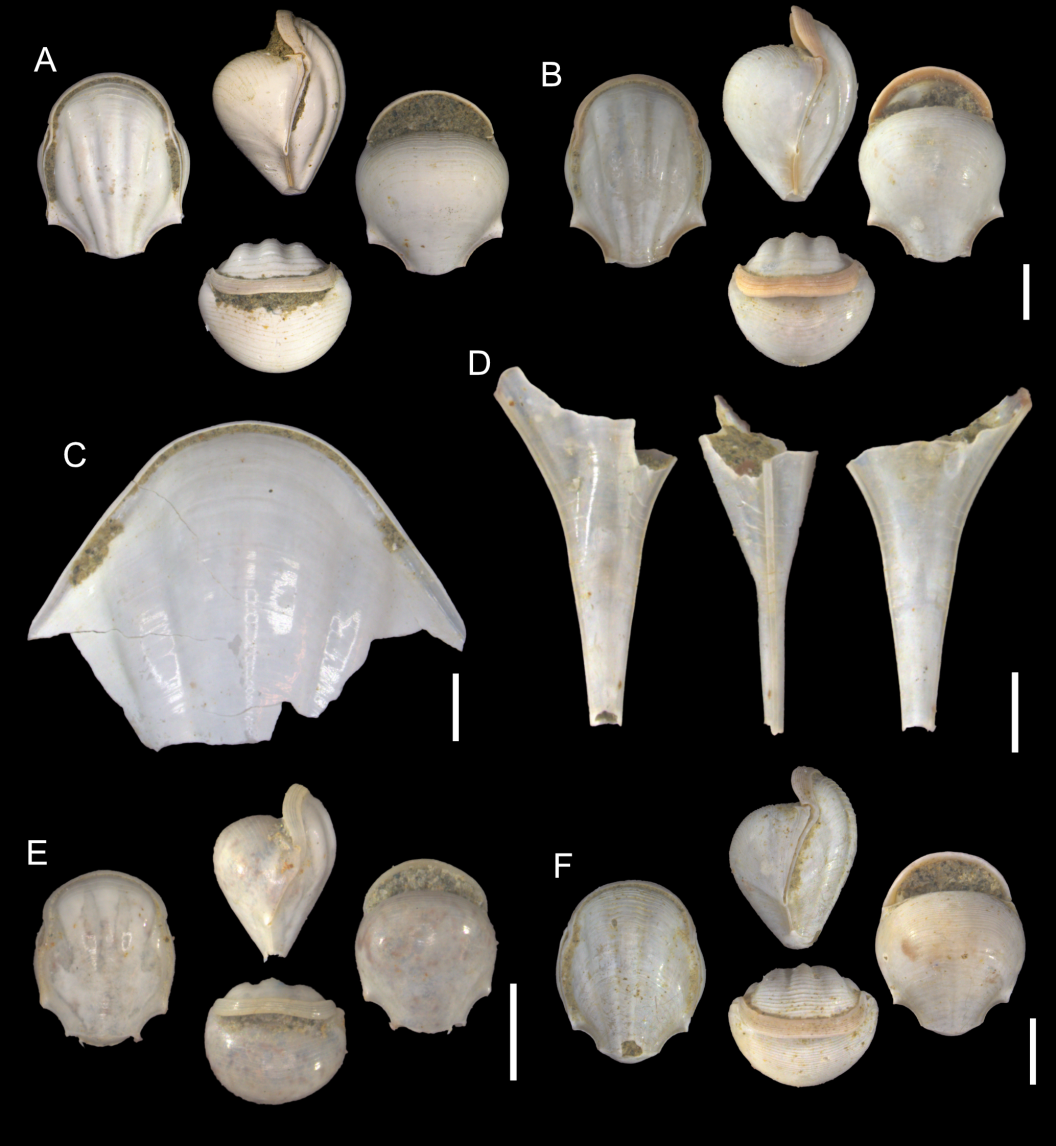
Species of the subfamily Diacriinae from the Late Pleistocene Szekou Formation, Taiwan. (A and B) *Diacria erythra* Van der Spoel, 1971, ASIZF0101141–142. (C and D) *Diacria trispinosa* (Blainville, 1821), ASIZF0101143–144. (E and F) *Telodiacria quadridentata* (Blainville, 1821), ASIZF0101145–146. Scale bars for A–B, C, D, E, F equal 1 mm.

Subfamily Diacriinae Rampal, 2019
Genus *Diacria* Gray, 1840
*Diacria erythra* Van der Spoel, 1971
(Figs. 8A–8B)
1971 *Diacria quadridentata erythra* forma *erythra*; Van der Spoel: 5, fig. 6.

**Description:** Shell medium in size (3.4 mm in SH, Table S3), rather higher than wide (2.7 in SW). Dorsal side moderately convex. Radial ribs thick and evident along dorsal side. Multiple transverse striae present closer to aperture. Thickened dorsal apertural margin brownish in color in inner and outer sides. Five dorsal radial ribs, three central ribs most distinct. Ventral side well-inflated, broader than dorsal. Ventral apertural lip small. Multiple transverse striae present closer to aperture margin. Apical sine absent, its base moderate in width. Lateral spines not recognizable due to lateral parts of the sell broken. Lines connecting apical spine with lateral spines slightly concave. Distance between lateral spine’s tips slightly larger than the greatest width of shell.

**Remarks:** Within the Szekou assemblage, *D. erythra* is relatively abundant (Table 1). This species closely resembles *Telodiacria quadridentata* Blainville, 1821 in shell morphology. However, it can be distinguished by its larger adult shells (Table S3) and by radial ribs that remain distinct up to the apical part of the shell.

The reported occurrences of this species remain somewhat uncertain. For instance, the only record from Pleistocene deposits in Japan, described as *Diacria quadridentata erythra* by Shibata & Ujihara (1983), likely represents *T. quadridentata* rather than *D. erythra*. Furthermore, many published records list the species without providing taxonomic descriptions or figures, potentially including additional misidentifications of *D. erythra* as *T. quadridentata*, or vice versa. In this context, our record of *D. erythra* from the Late Pleistocene of Taiwan represents the first confirmed fossil occurrence of this species in East and Southeast Asia.

**Table:** 

*Diacria trispinosa* (Blainville, 1821)
(Figs. 8C–8D)
1821 *Hyalaea trispinosa*; Blainville: 82.
1972 *Diacria bisulcata* Gabb, 1873; Noda: 478, pl. 57, fig. 18.
1979 *Diacria trispinosa* (Blainville, 1821); Shibata: pl. 20, figs. 41–46.
1986 *Diacria trispinosa* (Blainville, 1821); Ujihara: pl. 1, fig. 6.
1984 *Diacria trispinosa* forma *trispinosa* (Blainville, 1821); Shibata: 84, pl. 25, figs. 1–3.
1986 *Diacria trispinosa* forma *trispinosa* (Blainville, 1821); Shibata, Ishigaki & Ujihara: 48, pl. 8, figs. 7, 8.
1990 *Diacria trispinosa* forma *trispinosa* (Blainville, 1821); Ujihara, Shibata & Saito: 318, pl. 2, figs. 1–3.
1996 *Diacria trispinosa* forma *trispinosa* (Blainville, 1821); Ujihara: 781, figs. 6.7, 6.8.
2007 *Diacria trispinosa* forma *bisulcata* (Gabb, 1873); Janssen: 102, pl. 7, fig. 4; pl. 8, fig. 5; pl. 25, figs. 2, 3.
2012 *Diacria trispinosa* forma *bisulcata* (Gabb, 1873); Janssen & Grebneff: 24, figs. 17a, 17b.

**Description:** Shell big, lozenge in form with elongated caudal part. Dorsal side slightly inflated, shiny, with five radial ribs. Central tree ribs relatively prominent, almost equal in width. Central rib with slightly elevated ridge in the middle. Lateral ribs flatten towards lateral spines. Dorsal apertural margin thickened, arch-shaped. Ventral part absent in examined specimens. Lateral spines missing. Apical spine long, flattened.

**Remarks:** In our examined samples, this species is rare and represented by only a few poorly preserved specimens (Table 1). However, at least one specimen, preserving the dorsal part of the shell (Fig. 8C), displays diagnostic features sufficient for reliable identification. The remaining specimens consist of isolated caudal spines, whose morphology suggests they also belong to *D. trispinosa*.

*Diacria trispinosa* has been reported from multiple localities in Japan, including the Okinawa Islands (Noda, 1972), Kyushu (Ujihara, 1996), and as far north as the Boso Peninsula in central Honshu (*e.g.*, Shibata, 1979; Shibata, 1984; Shibata, Ishigaki & Ujihara, 1986). Its stratigraphic range extends from the Miocene to the Pleistocene. Outside Japan, fossil occurrences of *D. trispinosa* in the IWP are limited to Pliocene deposits in Luzon, Philippines (Janssen, 2007) and Buton, Fiji Archipelago (Janssen & Grebneff, 2012), where *D. trispinosa* forma *bisulcata* Gabb, 1873 has been reported.

This species has been described under two morphotypes, *Diacria trispinosa* forma *trispinosa* (Blainville, 1821) and *D. trispinosa* forma *bisulcata*, which have also been treated as subspecies or even distinct species by some authors. The main difference lies in the ribbing pattern: in *D. trispinosa* forma *bisulcata*, the three dorsal ribs are merged into a single central rib (Janssen, 2007). However, our preliminary re-examination of the published descriptions and figures suggests that some of these form-level identifications may be misinterpreted. For example, Shibata (1979), Shibata (1984) referred exclusively to *D. trispinosa* forma *trispinosa*, yet their illustrations appear to include specimens representing both forms. In our synonymy list, we include all these occurrences for completeness.

**Table:** 

Genus *Telodiacria* Rampal, 2019
*Telodiacria quadridentata* (Blainville, 1821)
(Figs. 8E–8F)
1821 *Hyalaea quadridentata*; Blainville: 81.

**Remarks:** See description for the Late Pleistocene *T. quadridentata* from Taiwan and related synonymy in Osipova & Lin (2025).

**Figure 9:**
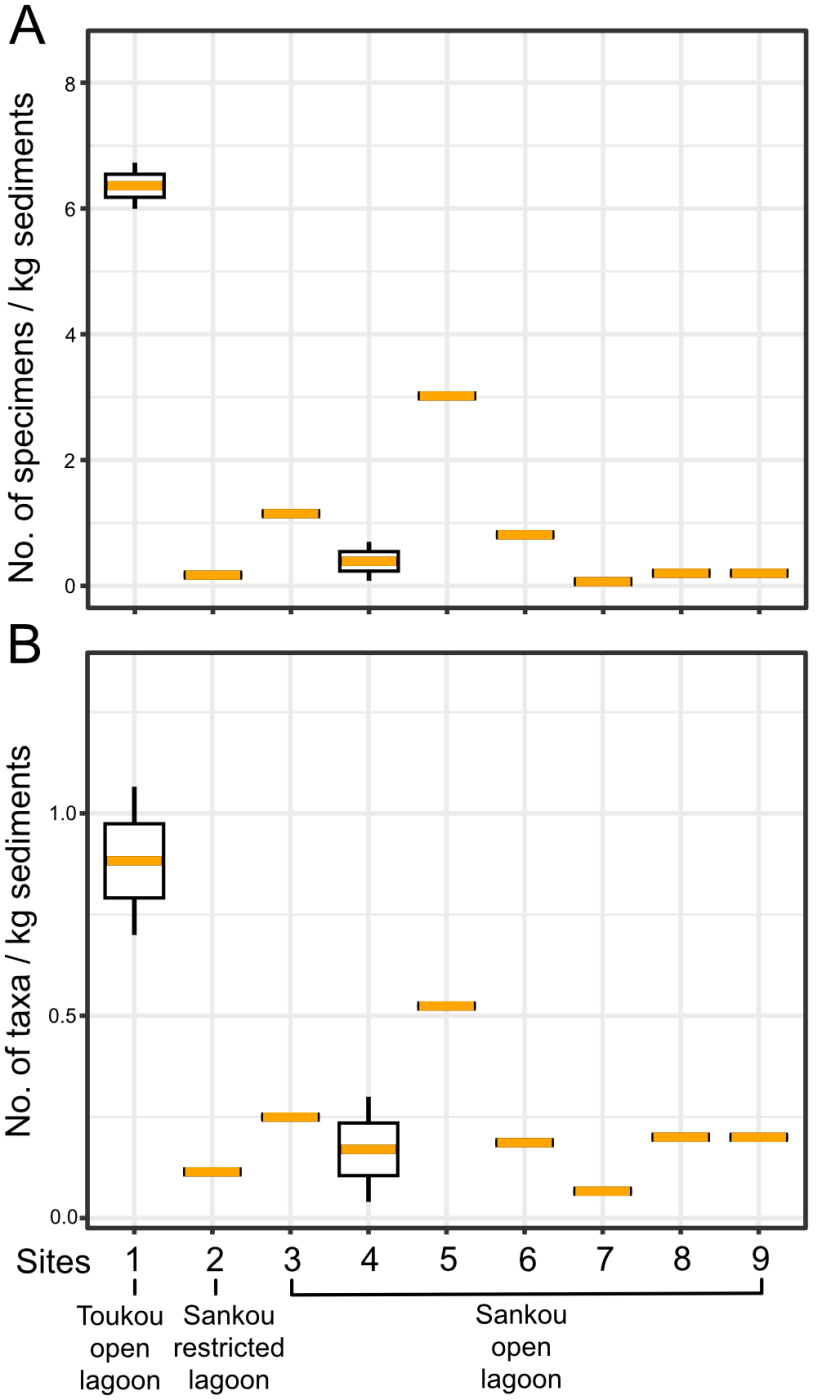
Differences in density and species richness of pelagic gastropods for three sampled localities. (A) Density of specimens; (B) pelagic gastropod species richness. Box plots with lower (25th percentile), median, and upper (75th percentile) boundaries.

### Paleobiodiversity of the Szekou pelagic gastropods

Examination of 69 bulk sediment samples from the Szekou Formation, representing different depositional environments (open and restricted lagoon) in two valleys (Toukou and Sankou), provided an opportunity to document and evaluate the diversity of pelagic gastropods previously unknown from this locality. Both major groups of shelled pelagic gastropods, heteropods and pteropods, were recovered. In total, 485 specimens representing 22 taxa were identified. The highest raw abundance and taxonomic richness were observed at Sankou open-lagoon sites (sites 3–9; Table 1), whereas the lowest values were recorded at the Sankou restricted-lagoon site (site 2).

Analysis at the level of individual sampling sites (Fig. 9) revealed no statistically significant differences in either specimen density (Kruskal–Wallis test: *χ*^2^ = 9.23, *df* = 8, *p* = 0.323) or taxonomic density (Kruskal–Wallis test: *χ*^2^ = 7.73, *df* = 8, *p* = 0.461) among the nine sites. Although raw values show considerable variability, including higher densities at one site (site 1, Toukou open lagoon), this variability is not consistently structured across sites. These results indicate that site-level heterogeneity does not drive the observed patterns.

**Figure 10:**
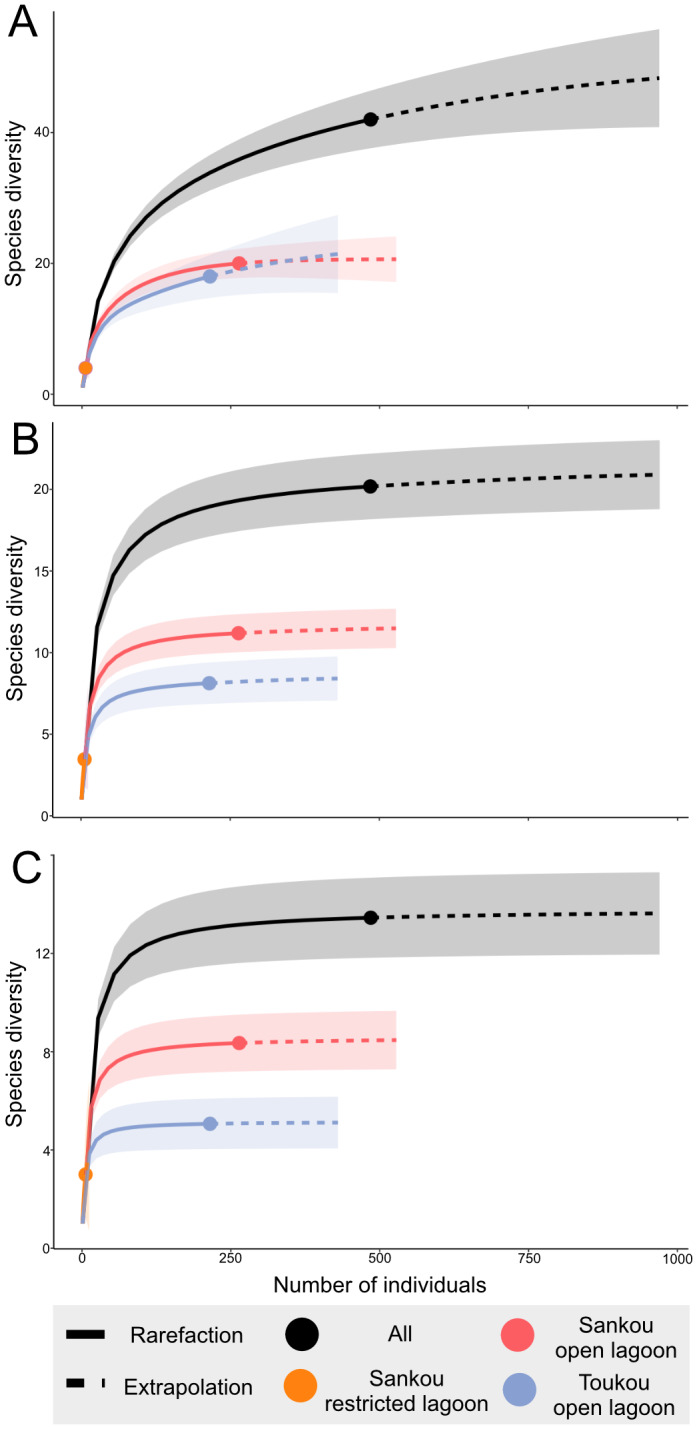
Rarefaction curves of species diversity (Hill numbers) among three sampling localities. (A) Species richness (0 D); (B) Shannon diversity (1 D); (C) Simpson diversity (2 D). Shaded areas represent 95% confidence interval for their respective sites based on 300 bootstrap replicates.

Rarefaction analyses revealed informative patterns across three diversity indices (Fig. 10). The species richness curve (^0^*D*; Fig. 10A) suggests that additional sampling would likely yield up to 40 taxa from the Szekou Formation. Rarefaction curves for open-lagoon assemblages generally showed higher diversity compared with the restricted-lagoon assemblage. Notably, despite differing sampling efforts between Toukou (*n* = 7) and Sankou open-lagoon sites (*n* = 55), their species richness was relatively similar. By contrast, when comparing sites with equal sampling effort (Toukou open-lagoon, *n* = 7; Sankou restricted-lagoon, *n* = 7), species richness and abundance (Table 1), and species diversity (Fig, 10A) were substantially lower in the restricted-lagoon assemblage. Rarefaction curves for Shannon (^1^*D*) and Simpson (^2^*D*) diversity indices approached asymptotes, indicating adequate sampling coverage for abundant and dominant taxa. Approximately seven abundant taxa were inferred for the Toukou open-lagoon assemblage and 11 for the Sankou open-lagoon assemblage (Fig. 10B). Similarly, dominant taxa were estimated at roughly five for Toukou and eight for Sankou open-lagoon sites (Fig. 10C).

### Assemblage comparison

**Figure 11:**
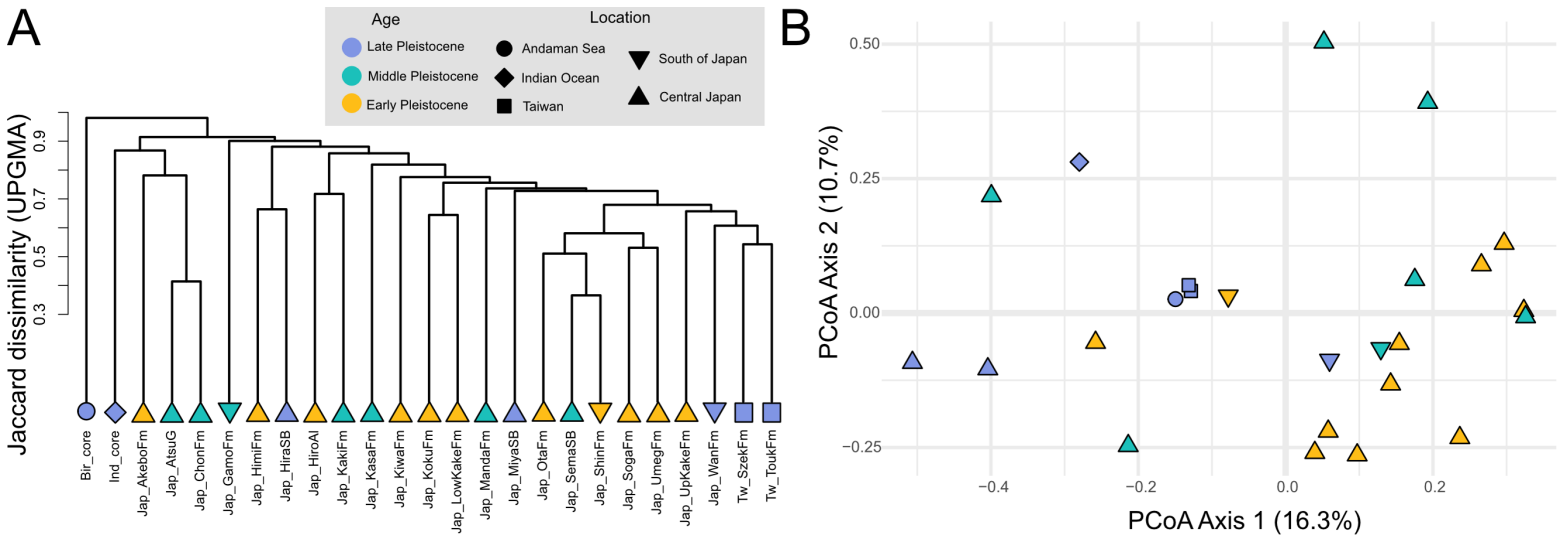
Similarity relationships among Pleistocene holoplanktonic gastropod assemblages from the Indo–West Pacific. (A) Cluster dendrogram based on presence–absence composition of assemblages using the Jaccard index and unweighted pair-group method with arithmetic mean (UPGMA); (B) principal coordinate analysis (PCoA) of the same presence–absence data based on the Jaccard index, showing the first two axes.

Hierarchical clustering based on the Jaccard dissimilarity matrix and the UPGMA method did not reveal any clear grouping patterns related to either age or locality (Fig. 11A). Despite the relatively large number of assemblages analyzed from Japan (Table S5), these did not form coherent clusters corresponding to specific regions or time intervals. The two assemblages from Taiwan cluster together; however, given the limited number of Taiwanese localities, this pattern should be interpreted with caution and cannot be considered robust evidence for regional differentiation. PCoA (Fig. 11B) further highlights substantial overlap among assemblages, with no strong separation along either age or geographic gradients. Overall, both clustering and ordination analyses indicate weak spatial and temporal structuring of Pleistocene holoplanktonic gastropod assemblages across the Indo–West Pacific.

ANOSIM and ISA were conducted using three predefined age groups and four locality groups. Within age groups, assemblages shared many taxa but showed moderate separation (Global *R* = 0.2429, *p* = 0.002, 999 permutations). ISA (Table 2) identified two pteropod taxa, *T. quadridentata* (IndVal = 0.676) and *H. inflata* (IndVal = 0.655), as indicators of the Late Pleistocene. When comparing across locality groups, differences were almost the same (Global *R* = 0.3312, *p* = 0.029, 999 permutations). Nevertheless, ISA identified three taxa, a heteropod *A. turriculata* (IndVal = 0.951) and pteropods *D. bandaensis* and *D. longirostris* (both IndVal = 1.0), as being most strongly associated with the Taiwanese assemblages. Conversely, *S. subula* (IndVal = 0.944) was broadly associated with assemblages from all regions except central Japan.

**Table 2:** Indicator Species Analysis of holoplanktonic gastropod taxa across age and locality groups.

	**Taxon**	**IndVal**	**p-value**
***Age group***			
Late Pleistocene	*Telodiacria quadridentata*	0.676	0.029
Late Pleistocene	*Heliconoides inflata*	0.655	0.034
***Locality group***			
Taiwan	*Atlanta turriculata*	0.951	0.047
Taiwan	*Diacavolinia bandaensis*	1	0.008
Taiwan	*Diacavolinia longirostris*	1	0.008
Andaman Sea + South of Japan + Taiwan	*Styliola subula*	0.944	0.02

## Discussion

### Taxonomic composition and paleoecological significance

Our assessment of holoplanktonic gastropod diversity and abundance in the Late Pleistocene sediments of southern Taiwan provides new data on the distribution and occurrence of both heteropods and pteropods in the IWP region. Previous reports of holoplanktonic gastropods from Taiwan were limited to several Oligocene records (Cheng, 1981; Chen & Huang, 1990) and to Late Pleistocene occurrences from northern Taiwan (Osipova & Lin, 2025). Most of the taxa identified in this study represent the first fossil records of holoplanktonic gastropods from southern Taiwan, including five heteropod taxa and eight pteropod taxa.

Analysis of richness and density across the Szekou Formation allowed us to evaluate the distribution of holoplanktonic gastropods within different depositional environments. Previous studies of other molluscan groups from this formation demonstrated differences in taxonomic composition between restricted and open lagoon settings (Chen et al., 1991). In contrast, our results show no statistically significant differences between these two environments for holoplanktonic gastropods, although raw estimates indicate lower richness and density in the restricted lagoon. This apparent discrepancy reflects uneven sampling effort and high within-environment variability, such that trends visible in raw values do not translate into statistically significant differences. It is worth noting, differences in sedimentation rates and dilution effects (Kidwell, 1986) between open- and restricted-lagoon settings can contribute to the observed variability in specimen and taxon densities. However, the lack of statistically significant differences among sites and environments suggests that any such effects did not generate a consistent signal strong enough to structure the assemblages analyzed here.

Rarefaction analysis suggests that additional sampling would likely yield up to 40 taxa. This pattern is consistent with the general biogeographic trend observed in modern holoplanktonic gastropods, which show high diversity but relatively low abundance in lower latitudes (Angel, 1997; Janssen & Peijnenburg, 2017). The low intraspecific morphological variability in some taxa (*e.g.,* Van der Spoel, Bleeker & Kobayasi, 1993; Wall-Palmer et al., 2016), combined with the potential loss of delicate diagnostic features during fossilization (Janssen & Peijnenburg, 2017; Hart et al., 2020), suggests that certain species may have been under-identified. Overall, our results indicate that the Late Pleistocene holoplanktonic gastropod assemblage of southern Taiwan was characterized by a community structure with relatively few highly dominant species and a larger number of rare species, a pattern broadly consistent with that observed in modern pelagic gastropod assemblages (Bé & Gilmer, 1977; Pierrot-Bults & Peijnenburg, 2015; Wall-Palmer et al., 2016; Wall-Palmer et al., 2018).

### Relations of IWP assemblages

Understanding the structure of pelagic gastropod assemblages in the IWP region is key to the reconstruction of past distribution patterns and understanding the evolutionary and ecological history of these taxa. Bernasconi & Robba (1982) compiled data from available sources and proposed potential dispersal pathways for pteropods, highlighting that the distribution of thecosomatous pteropods traceable to the present was established during the Pliocene–Pleistocene transition. A similar pattern has been proposed for atlantid heteropods (Wall-Palmer et al., 2016). During this time, pteropod distribution was strongly controlled by climate-related factors, including sea surface temperature, salinity, water-column stratification, and ocean circulation (Bernasconi & Robba, 1982; Janssen & Peijnenburg, 2017; Hart et al., 2020; Peijnenburg et al., 2020). However, heteropods are considered to be less sensitive to changes in temperatures and environmental conditions during that time (Van der Spoel, 1972; Wall-Palmer et al., 2014).

Fossil records of Pleistocene pelagic gastropods in the IWP are largely restricted to Japan, likely reflecting sampling bias, as most studies were conducted in Japan during the mid-to-late 20th century. Other known occurrences are limited to sediment core samples from the Andaman Sea (Sijinkumar, Nath & Guptha, 2010) and the Indian Ocean (Wall-Palmer et al., 2014), followed by a recently recovered assemblage from Taiwan (Osipova & Lin, 2025). The present study supplements previously published records with new data from Taiwan and compares these with Pleistocene assemblages from other IWP localities.

Our analysis reveals low clustering and no evident spatiotemporal pattern of Pleistocene holoplanktonic gastropods in the IWP (Fig. 11). It correlates well with the previous study of Late Pleistocene assemblages from the Caribbean and Indian regions (Wall-Palmer et al., 2014). That study showed that little change in species composition, species richness, and abundance was observed among those localities. The authors suggested that the low latitude location resulted in the low temperature variation despite the changes of glacial and interglacial periods (temperature variations in the Caribbean and Indian Ocean were within 1–3 °C between glacial-interglacial periods, while in the Mediterranean this variation was 4–6 °C). Likewise, the subtropical location of our studied IWP sites probably provided a warm-water realm for the holoplanktonic gastropods at that time that resulted in a more cosmopolitan distribution and species composition in these latitudes.

At the same time, in a bit higher latitude, a biogeographical pattern was detected during the Cenozoic for bivalves (Chinzei, 1986; Ogasawara, 1994), separating Japan into four zones. This zonation was maintained during the Quaternary, with subtropical extending up to the central Honshu, warm-temperate climate prevailing in the Korea Strait, and mid-temperate climate zone covering the eastern and western parts of the central Honshu. This must be true not only for benthic mollusks but also for pelagic ones. Wall-Palmer et al. (2014) noted that in the Mediterranean Sea, compared to tropical sites, the more evident changes are observed in assemblage composition that might reflect a considerable variation in temperatures.

Nevertheless, only weakly separated patterns can be seen among the studied assemblages (Fig. 11B). Hierarchical clustering shows both age-consistent and mixed-age groupings among Japanese assemblages. While some Early and Middle Pleistocene assemblages cluster with others of similar age, clusters comprising assemblages of different ages are equally present, indicating that age alone does not strongly structure assemblage similarity. Unlike the Mediterranean basin, which was a permanent semi-enclosed system from the Late Miocene till the Holocene (*e.g.*, Karami et al., 2011; Meijer, 2021), the IWP region was subjected to variations in ocean circulation, particularly intensification of the Kuroshio Current during stronger glacial–interglacial cycles around 1 Ma (Gallagher et al., 2015), which likely influenced pelagic gastropod distribution. It is still possible that the active changes in the ocean circulation acted in favor of a wider distribution of pelagic gastropods, leading to the spatially and temporally poorly clustered assemblages.

Several species showed weak yet present association with specific age groups or localities. The pteropods *T. quadridentata* and *H. inflata* are indicators of the Late Pleistocene across most studied localities. Shibata (1986) noted that these species first appeared in central Japan during the Late Pleistocene, although *H. inflata* is already present in Pliocene deposits of the Philippines (Janssen, 2007), suggesting a later arrival in Japan. Regarding geographic distribution, *S. subula* showed the strongest association with assemblages from the Andaman Sea, Taiwan, and southern Japan. This agrees with its modern distribution, where it is present but less common in mid-latitude Japanese waters (Bé & Gilmer, 1977). The exclusive association of *A. turriculata*, *D. bandaensis*, and *D. longirostris* with the Taiwanese assemblage likely reflects misinterpretations of these species in previous publications (as discussed in the Remarks section), which may have resulted in their stronger linkage to Taiwan in our analysis. Thus, we do not consider these results to represent their true geographic affiliation during the Pleistocene.

Our study adds new data to the sparse record of Pleistocene holoplanktonic gastropods from the IWP, but significant gaps remain. The limited number of localities, particularly outside Honshu, hampers our ability to fully resolve biogeographic and temporal patterns. Additional reference material, especially from early Quaternary and Neogene deposits, is needed to clarify the timing of first appearances and regional faunal turnovers. Improved dating of existing sites would strengthen interregional comparisons and allow a finer correlation with paleoceanographic events. Future research should also expand the temporal scope beyond the Pleistocene, integrating Neogene assemblages to reconstruct longer-term evolutionary and biogeographic trends. Broader geographic coverage and standardized sampling will be essential to refine our understanding of how pelagic gastropod communities responded to climatic and oceanographic changes across the IWP.

## Conclusions

This study provides the first comprehensive record of Late Pleistocene holoplanktonic gastropods from southern Taiwan, extending the known range of both heteropods and pteropods in the IWP. The assemblage from the Szekou Formation, comprising eight heteropod and 14 pteropod taxa, shows no significant differences in richness or abundance between open and restricted lagoonal settings, suggesting that pelagic taxa were less affected by local environmental conditions than benthic mollusks, at the same time showing a relatively balanced community. Comparison with other Pleistocene IWP localities reveals weak spatial and temporal clustering, implying that subtropical waters and stable warm conditions, coupled with intensified Kuroshio circulation, promoted cosmopolitan distributions during glacial–interglacial cycles. While these findings fill an important geographic gap in the regional record, broader sampling and improved chronological control are needed to reconstruct long-term biogeographic and paleoceanographic patterns of holoplanktonic gastropods in the Cenozoic IWP.

## Supplemental Information

10.7717/peerj.21046/supp-1 Supplemental Information 1 Location of sampling sites and sampling effort at the Szekou Formation

10.7717/peerj.21046/supp-2 Supplemental Information 2 Number of specimens recovered from each sampling site

10.7717/peerj.21046/supp-3 Supplemental Information 3 Shell measurements and number of specimens of fossil holoplanktonic mollusk NA means the feature cannot be measured. Dash means the feature was not measured in the specimen. The tilde symbol represents the average value (for multiple specimens).

10.7717/peerj.21046/supp-4 Supplemental Information 4 List of records of Pleistocene holoplanktonic gastropods from the Indo–West Pacific

10.7717/peerj.21046/supp-5 Supplemental Information 5 List of localities considered in the study

## Additional Information and Declarations

### Field Study Permissions

The following information was supplied relating to field study approvals (i.e., approving body and any reference numbers): No permit needed.

### Data Availability

The following information was supplied regarding data availability: The GPS coordinates of each examined locality, measurements of analyzed material and the raw taxa list and localities used for analyses are available in the Supplemental Files.
